# Inflammatory signals are sufficient to elicit TOX expression in mouse and human CD8^+^ T cells

**DOI:** 10.1172/jci.insight.150744

**Published:** 2021-07-08

**Authors:** Nicholas J. Maurice, Jacqueline Berner, Alexis K. Taber, Dietmar Zehn, Martin Prlic

**Affiliations:** 1 Vaccine and Infectious Disease Division, Fred Hutchinson Cancer Research Center, Seattle, Washington, USA.; 2 Molecular and Cellular Biology Graduate Program, University of Washington, Seattle, Washington, USA.; 3 Division of Animal Physiology and Immunology, Technical University of Munich School of Life Sciences Weihenstephan, Technical University of Munich, Freising, Germany.; 4 Department of Immunology, University of Washington, Seattle, Washington, USA.

**Keywords:** Immunology, Inflammation, T cells

## Abstract

T cell receptor (TCR) stimulation leads to the expression of the transcription factor thymocyte selection–associated high-mobility group box (TOX). Prolonged TCR signaling, such as encountered during chronic infections or in tumors, leads to sustained TOX expression, which is required for the induction of a state of exhaustion or dysfunction. Although CD8^+^ memory T (Tmem) cells in mice typically do not express TOX at steady state, some human Tmem cells express TOX but appear fully functional. This seeming discrepancy between mouse and human T cells has led to the speculation that TOX is differentially regulated between these species, which could complicate the interpretation of preclinical mouse model studies. We report here that, similar to TCR-mediated signals, inflammatory cytokines are also sufficient to increase TOX expression in human and mouse Tmem cells. Thus, TOX expression is controlled by the environment, which provides an explanation for the different TOX expression patterns encountered in T cells isolated from specific pathogen–free laboratory mice versus humans. Finally, we report that TOX is not necessary for cytokine-driven expression of programmed cell death 1. Overall, our data highlight that the mechanisms regulating TOX expression are conserved across species and indicate that TOX expression reflects a T cell’s activation state and does not necessarily correlate with T cell dysfunction.

## Introduction

T cell exhaustion (i.e., dysfunction) is driven by chronic T cell receptor (TCR) stimulation with cognate antigen (Ag; refs. 1–3). It describes a differentiation state in which T cells have diminished capacity to respond to stimulatory inputs and limited effector capacity (2–4). The purpose of T cell exhaustion during chronic infections may be to limit tissue pathologies when pathogen cannot be immunologically eliminated (5, 6). Though exhaustion could be considered an immunologic concession during chronic infection, it also occurs in tumors and causes an attenuated antitumor cytotoxic T cell response (7). Thus, mechanistically understanding and therapeutically overcoming T cell exhaustion has been a major goal of tumor immunotherapy. Chronic TCR stimulation elicits a program that leads to constitutively high expression of programmed cell death 1 (PD-1; ref. 8). PD-1 is an inhibitory receptor that is expressed by activated and exhausted T cells and is often used as a biomarker to infer T cell functionality (9). When bound to its ligands, PD-1 negatively regulates T cell function (2). Therapeutic targeting of PD-1 with monoclonal antibodies, also referred to as immune checkpoint inhibitors, can reinvigorate a subset of these PD-1–expressing T cells (2, 10–12).

A set of recent studies demonstrated that the transcription factor thymocyte selection–associated high-mobility group box (TOX) protein drives or stabilizes this TCR-mediated T cell dysfunction and PD-1 upregulation (6, 13–16). When stably expressed, TOX drives Ag-specific T cell exhaustion in mouse models of chronic lymphocytic choriomeningitis virus (LCMV) infection, transplantable B16 melanoma, and inducible hepatocellular carcinoma (6, 13, 14). Further, putative tumor Ag–specific CD8^+^ T cells isolated from primary human breast, ovarian, and skin cancer samples, as well as those specific for hepatitis C virus (HCV), mirror this phenotype, suggesting TOX dictates exhaustion programs in humans, too (6, 13, 14). Of note, TOX expression by HCV-specific T cells is reduced after treatment and clearance of the infection, but it is still detectable at higher levels than in T cells from HCV infections that spontaneously resolve and among T cells specific for influenza A virus (IAV; ref. 6). Mechanistic insight is provided by targeted deletion of TOX in Ag-specific cytotoxic T cells, which diminishes PD-1 expression and restores functionality at the expense of cell survival (6, 13). Therefore, TOX concedes activation and effector function for exhaustion (i.e., PD-1 expression) and T cell survival during chronic TCR stimulation. In instances of brief TCR engagement, TOX is transiently induced to a level lower than that of exhausted T cells, but with limited known functional consequence (6, 13, 14).

Although the requirement for TOX has been well defined in the context of TCR-mediated dysfunction, there is nascent evidence that TOX expression by itself is not indicative of T cell exhaustion. Recent studies illustrated that TOX expression is detected in some functional CD8^+^ memory T (Tmem) cells, for instance, in CD8^+^ effector memory (Tem) and CD45RA-expressing Tem (Temra) subsets (17). CD8^+^ Tmem cells specific for the latent viruses, CMV and EBV, had elevated TOX expression, compared with those specific for acute infections, which further suggests that TCR signals are critical in regulating TOX expression (17). In a second study, it was shown that a fraction of the human Tmem population expresses *TOX* transcripts among other signature genes typically associated with T cell exhaustion (18). The observation that functional human Tmem cells express TOX also led to questions of whether TOX is functionally conserved between mouse and human T cells (19). Further complicating TOX and exhaustion, the murine tissue-resident Tmem (Trm) cell transcriptome is characterized by the concomitant expression of transcripts encoding *Tox*, exhaustion markers, TCR signaling components, and cytotoxic molecules, well after initial priming events (20, 21). Although the role of TOX in these TOX-expressing populations with and without signs of T cell exhaustion is not fully understood, these data suggest that TOX expression by Tmem cells cannot be reliably used to extrapolate T cell function.

Although the role of TCR signals in initiating and maintaining PD-1 and TOX expression has been well established, relatively little remains known about non-TCR signals that could regulate their expression in T cells (22). We considered that cytokine-mediated stimuli could also affect TOX expression levels without promoting the induction of T cell exhaustion. First, proinflammatory cytokines, such as IL-15, can induce PD-1 without agonist TCR signals (22). Second, Trm cells that are likely not detecting cognate Ag still upregulate PD-1 and other markers associated with exhaustion (20, 21, 23, 24), yet rely on IL-15 signaling for maintenance in some tissues (25, 26). Thus, inflammatory signals could provide an explanation for some of the seemingly disparate results of TOX expression and T cell function. Here, we show that proinflammatory cytokines were sufficient to induce TOX expression in the absence of agonist TCR signals in both mouse and human CD8^+^ Tmem cells while concurrently inducing the expression of cytotoxic molecules. Together, these data demonstrate that TOX expression per se does not indicate TCR-mediated dysfunction or even a recent TCR signals. We also demonstrate that PD-1 expression was still upregulated in TOX-deficient T cells, indicating that TOX was not necessary for PD-1 expression. Overall, our data reveal that TCR-independent mechanisms shape TOX and PD-1 expression heterogeneity in Tmem cells and indicate that they are conserved in both mouse and human T cells. Though these findings ultimately complicate the use of TOX exclusively as an exhaustion biomarker, they implicate TOX in inflammation-driven programs of Tmem cell activation.

## Results

### Cytokine stimulation induced TOX expression in murine CD8^+^ Tmem cells.

**Figure 1:**
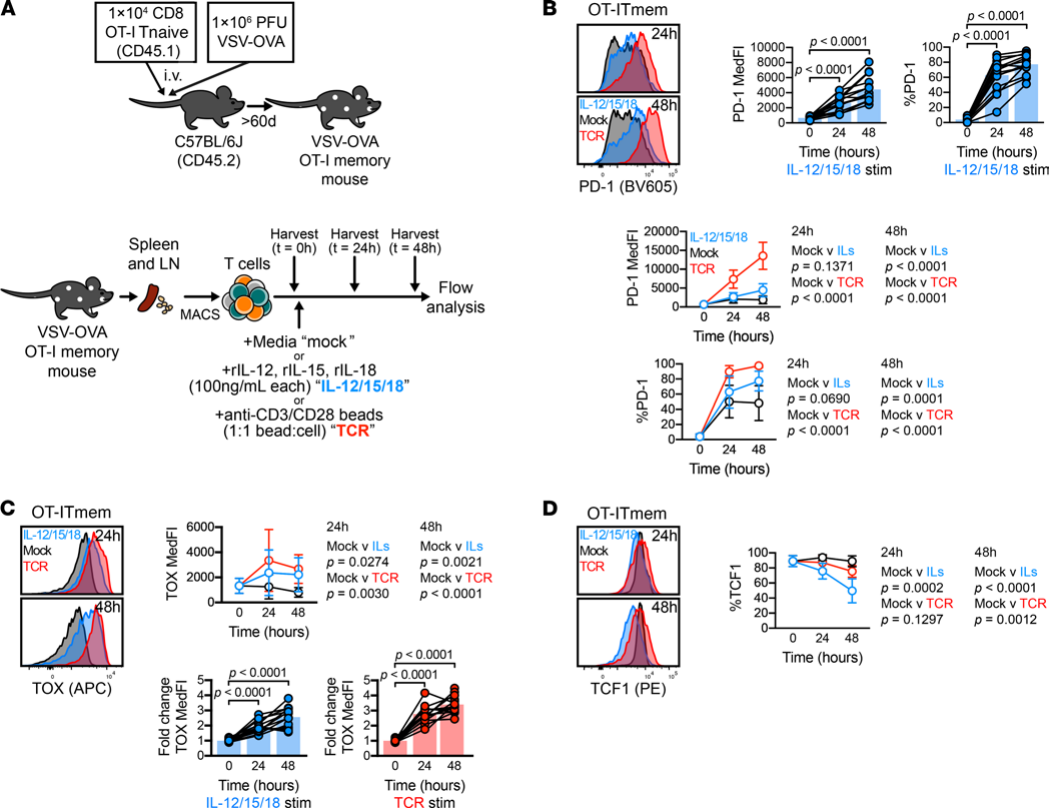
Cytokine stimulation induces TOX expression in murine CD8^+^ Tmem cells. (**A**) Schematic of OT-I memory mouse generation (top) and subsequent stimulation assays (bottom). OT-I Tnaive cells were transferred and expanded with VSV-OVA, then aged to stable memory contraction; after, T cells were enriched from VSV-OVA expanded OT-I memory animals and stimulated with media alone (mock), IL-12, IL-15, and IL-18 in combination (IL-12/15/18; each at 100 ng/mL), or anti-CD3/CD28 microbeads (TCR) at an approximately 1:1 bead/cell ratio. (**B** and **C**) expression of (**B**) PD-1, (**C**) TOX, and (**D**) TCF1 within stimulated OT-I Tmem cells throughout experiment time course. TOX MedFI fold change in **C** was calculated against average TOX MedFI from mock stimulations in a subset-specific, batch-specific, and time point–specific manner. In **B** and **C**, bar chart symbols represent 1 animal at a unique time point/condition and are connected by animal identity, with bar indicating mean; the indicated statistical significances in **B** and **C** were calculated using paired *t* tests. In **B**–**D**, symbols in line plots comparing stimulation conditions represent the mean across all animals for a specific time point/condition ± SD; the indicated statistical significances were calculated using Mann-Whitney *U* tests. Results from *n* = 14 mice across 7 experiments are shown in **B** and **C**. Results from *n* = 9 mice across 2 experiments are shown in **D**. TOX, thymocyte selection–associated high-mobility group box; Tmem, memory T cells; Tnaive, naive T cells; VSV-OVA, OVA-expressing vesicular stomatitis virus; PD-1, programmed cell death protein 1; MedFI, median fluorescence intensity.

The proinflammatory cytokines IL-12, IL-15, and IL-18 elicit IFN-γ and granzyme B (GzmB) expression in mouse and human CD8^+^ Tmem cells in the absence of agonist TCR signals (27–29). We first sought to determine if these cytokines could also induce TOX expression in a TCR-independent manner. To generate a well-defined population of CD8^+^ Tmem cells, we transferred congenically marked OT-I CD8^+^ T cells, which express a TCR specific for the SIINFEKL peptide of OVA, into WT C57BL/6J animals followed by infection with OVA-expressing vesicular stomatitis virus (VSV-OVA; Figure 1A). We waited 60 days or longer before using these mice for subsequent experiments (referred to as VSV-OVA OT-I memory mice; Figure 1A). We isolated T cells from the spleens and lymph nodes (LNs) from VSV-OVA OT-I memory mice using negative-selection magnet-activated cell sorting (MACS) prior to ex vivo stimulation experiments (Figure 1A). This was done to ensure that cytokines act directly on T cells (30). As a negative control, we cultured bulk T cells in media alone (mock), and as a positive control, we stimulated T cells with anti-CD3/CD28 microbeads (Figure 1A). We used a combination of rIL-12, rIL-15, and rIL-18 (IL-12/15/18) to induce IFN-γ and GzmB expression in a TCR-independent manner (Figure 1A). We found that IL-12/15/18 stimulation induced PD-1 expression in OT-I Tmem cells, but the increase in expression was markedly higher after TCR ligation (Figure 1B). PD-1 frequency and median fluorescence intensity (MedFI) in OT-I Tmem increased throughout the duration of IL-12/15/18 stimulation (Figure 1B). Similarly, TCR and IL-12/15/18 stimulation induced TOX upregulation in OT-I Tmem cells (Figure 1C). Next, we measured TCF1 expression, a transcription factor needed for Tmem self-renewal that is lost in terminally exhausted Tmem cells (31–34). Alongside increasing PD-1 and TOX levels, both TCR-mediated and IL-12/15/18–mediated stimulation led to significant loss of TCF1 expression in OT-I Tmem cells (Figure 1D). In sum, these data indicate that phenotypes often associated with exhaustion can be induced by TCR-independent, cytokine-mediated Tmem activation. Finally, we sought to determine whether stimulation similarly affected endogenous CD8^+^ Tmem and CD8^+^ naive T (Tnaive) cells. IL-12/15/18 stimulation significantly increased TOX expression in endogenous CD8^+^ Tmem cells but was not observed to the same degree in CD8^+^ Tnaive cells (Supplemental Figure 1, A and B; supplemental material available online with this article; https://doi.org/10.1172/jci.insight.150744DS1). This CD8^+^ Tmem cell–specific response is, too, reflected in IL-12/15/18–mediated upregulation of PD-1 (Supplemental Figure 1, C and D). This is likely, in some degree, due to the different propensities of T cell subsets (both major and memory) to become efficiently activated by cytokines (35) and differences in cytokine receptor expression (particularly Tnaive cells, which require TCR-mediated activation to induce IL-12R and strongly increase IL-18R expression; refs. 36–38). Much akin to OT-I Tmem cells, TCR stimulation dramatically increased both TOX MedFI and PD-1 expression across endogenous subsets (Supplemental Figure 1, A–D), though the fold change in TOX staining intensity was most pronounced in CD8^+^ Tmem cells (Supplemental Figure 1A). Though IL-12/15/18 stimulation increased TOX MedFI in transgenic and endogenous CD8^+^ Tmem cells, it was initially to a lower degree than that of TCR-stimulated cells (Figure 1C and Supplemental Figure 1A). Because short-term TCR and IL-12/15/18 stimulation could dramatically augment TOX and PD-1 expression in CD8^+^ Tmem cells from VSV-OVA OT-I memory mice, we next sought to test if the upregulation of TOX and PD-1 compromises functionality.

### Functional CD8^+^ Tmem cells expressed TOX, PD-1, and effector proteins.

**Figure 2:**
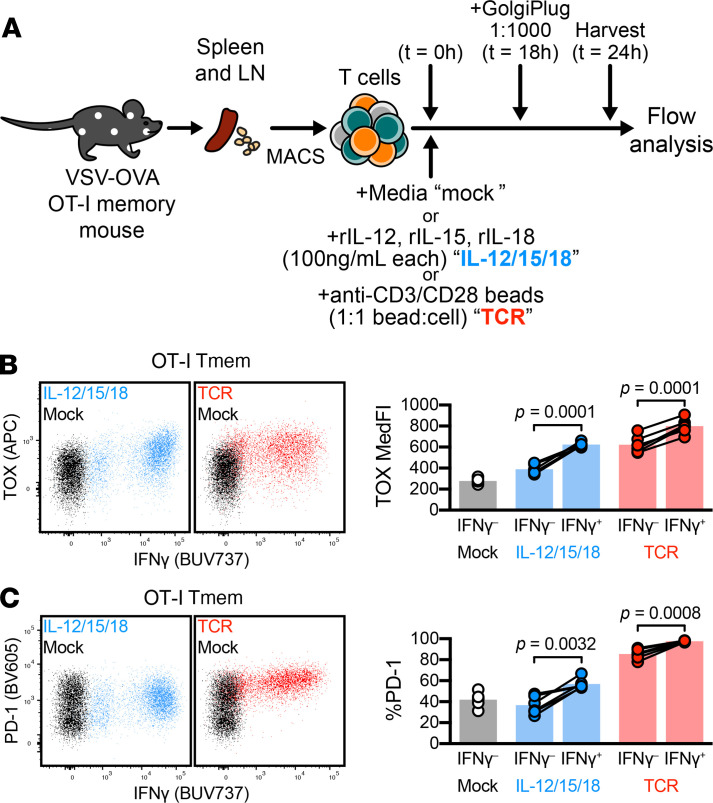
TOX and PD-1 expression occur in functional CD8^+^ T cells. ICS in tandem with TOX interrogation. (**A**) Experiment schematic, in which bulk T cells from VSV-OVA OT-I memory mice were stimulated (mock, black; IL-12/15/18, blue; TCR, red). Cells were treated with GolgiPlug 18 hours into stimulation and harvested for flow staining and analysis at 24 hours. (**B** and **C**) Expression of (**B**) TOX and (**C**) PD-1 in IFN-γ^+^ and IFN-γ^–^ OT-I Tmem cells. Representative plots depict cells from the same animal across different stimulation conditions. Symbols in **B** and **C** represent a T cell population within a unique animal with symbols connected by animal identity (*n* = 6 across 2 experiments). Bars represent mean and indicated statistical significances were calculated by paired *t* tests. TOX, thymocyte selection–associated high-mobility group box; ICS, intracellular cytokine staining; Tmem, memory T cells; VSV-OVA, OVA-expressing vesicular stomatitis virus; PD-1, programmed cell death protein 1; TCR, T cell receptor.

We isolated T cells from VSV-OVA OT-I memory mice, as outlined in Figure 1. We stimulated T cells in the presence of Golgi inhibitors (Figure 2A) and found that OT-I Tmem cells produced substantial amounts of IFN-γ after IL-12/15/18 or TCR stimulation; yet IFN-γ–expressing OT-I Tmem cells demonstrated higher TOX and PD-1 expression than those that failed to make IFN-γ (Figure 2, B and C). Similarly, OT-I Tmem cells that produced GzmB after stimulation also demonstrated increased TOX and PD-1 expression (Supplemental Figure 2, A and B). Together, these data indicate that TOX and PD-1 expression levels were elevated in activated, functional CD8^+^ Tmem cells and suggest that TOX expression was also part of a cytokine-driven T cell activation program.

### Induction of TOX and PD-1 was heterogeneous in CD8^+^ Tmem cells.

**Figure 3:**
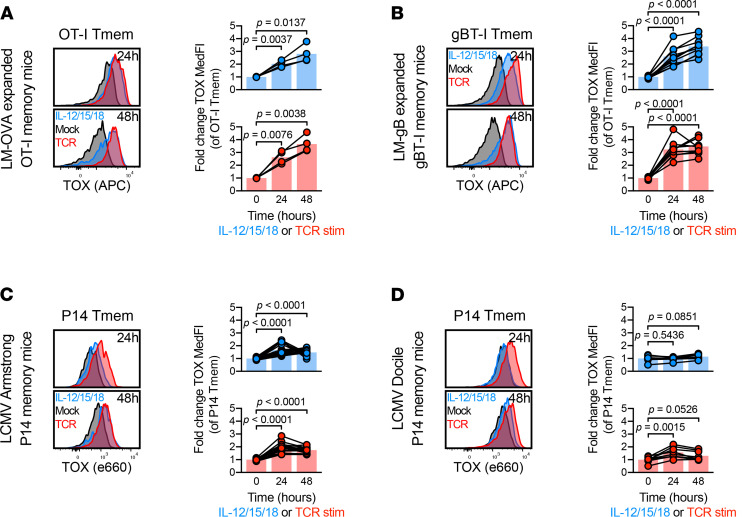
Cytokine-mediated TOX induction is limited in exhausted T cells. (**A** and **B**) Changes in TOX expression within *L*. *monocytogenes*–expanded TCR-transgenic Tmem cells: OT-I, specific for OVA Ag and gBT-I, specific for gB Ag. MACS-enriched T cells from *L*. *monocytogenes–*expanded OT-I or gBT-I memory mice were stimulated with media alone (mock), recombinant IL-12, IL-15, and IL-18 in combination (IL-12/15/18; each at 100 ng/mL), or anti-CD3/CD28 microbeads (TCR) at a approximately 1:1 cell/bead ratio. (**A** and **B**) Representative TOX expression and TOX MedFI fold change during stimulation in *L*. *monocytogenes–*primed (**A**) OT-I and (**B**) gBT-I Tmem cells. (**C** and **D**) Changes in TOX expression within LCMV-specific TCR-transgenic P14 T cells expanded by acute (Armstrong) or chronic (Docile) LCMV infection. (**C** and **D**) Representative TOX expression and TOX MedFI fold change during stimulation in P14 T cells primed by (**C**) LCMV Armstrong and (**D**) LCMV Docile. TOX MedFI fold change was calculated against average TOX MedFI within mock stimulation in a batch-specific, time point–specific manner. We calculated indicated statistical significances using paired *t* tests. Each symbol represents a sample at a unique time point/condition, with bars delineating mean, which are connected by donor (*n* = 4 *L*. *monocytogenes–*OVA expanded OT-I memory mice across 2 experiments; *n* = 10 *L*. *monocytogenes–*gB expanded gBT-I memory mice across 2 experiments; *n* = 17 LCMV Armstrong-expanded P14 memory mice across 4 experiments; *n* = 8 LCMV Docile-expanded P14 memory mice across 2 experiments). Mouse identities are consistent between representative flow plots within the same generation/adoptive transfer condition. TOX, thymocyte selection–associated high-mobility group box; Tmem, memory T cells; LCMV, lymphocytic choriomeningitis virus; MACS, magnet-activated cell sorting; PD-1, programmed cell death protein 1; MedFI, median fluorescence intensity; TCR, T cell receptor; gB, glycoprotein B; Ag, antigen.

To ensure that our data were not solely reliant on OT-I T cells, we also generated gBT-I memory mice using gBT-I TCR-transgenic cells (specific for an epitope of the herpes simplex virus 2 [HSV2] glycoprotein B [gB] protein) and a recombinant, gB epitope–expressing *Listeria* *monocytogenes* strain (*L*. *monocytogenes–*gB; Supplemental Figure 3B). After stable contraction of TCR-transgenic Tmem cells (≥60 d), we conducted stimulation assays as previously outlined (Figure 1A). IL-12/15/18–mediated or TCR-mediated stimulation led to comparable TOX upregulation in OT-I and gBT-I Tmem cells (Figure 3, A and B). Similarly, PD-1 expression was comparable in OT-I and gBT-I Tmem cells after stimulation (Supplemental Figure 3, C and D), with a concurrent loss of TCF1 expression (Supplemental Figure 3, E and F). We next asked if altering the nature of the priming infection could affect the ability to express TOX in response to cytokine-mediated activation at the memory stage. We adoptively transferred P14 transgenic T cells, a TCR-transgenic cell line specific for LCMV gp33, followed by infection with LCMV Armstrong or Docile (Supplemental Figure 3, G and H). These LCMV strains elicit acute and chronic infections, respectively (the latter causing T cell dysfunction). We then stimulated T cells (with the same culture setup as outlined in Figure 1A) from these P14 memory mice. P14 Tmem cells from LCMV Armstrong–infected mice readily upregulated PD-1 after TCR or IL-12/15/18 stimulation (Supplemental Figure 3I). The exhausted P14 Tmem cells from LCMV Docile–infected mice already uniformly expressed PD-1 prior to stimulation, but IL-12/15/18 or TCR stimulation further increased surface PD-1 expression (via increased MedFI; Supplemental Figure 3J). P14 Tmem cells from LCMV Armstrong–infected mice increased TOX expression after TCR or IL-12/15/18 stimulation (Figure 3C). However, exhausted P14 Tmem cells from LCMV Docile–infected mice significantly increased TOX expression only after TCR stimulation (Figure 3D). Overall, P14 Tmem cells from LCMV Docile–infected mice showed a much more limited fold change in TOX MedFI compared with P14 Tmem cells from LCMV Armstrong–infected mice (Figure 3C vs. Figure 3D). Although differences between CD8^+^ Tmem cells from mice with acute and chronic infections are expected, the differences between gBT-I and OT-I (~3- to 4-fold increase in TOX expression) compared with P14 (up to ~2-fold) need to be interpreted with caution because the gBT-I/OT-I and P14 experiments used different TOX antibody clones (REA473 and TXRX10, respectively). Overall, our data indicate that Tmem cells that were generated by different acute infections increased TOX expression in response to proinflammatory cytokines, suggesting that this was a broadly applicable mechanism of TOX induction in the Tmem cell compartment. We next sought to determine if PD-1 and TOX upregulation in response to stimulation was similarly recapitulated in human CD8^+^ T cells.

### Cytokine stimulation induced TOX and PD-1 in human Tmem cells.

**Figure 4:**
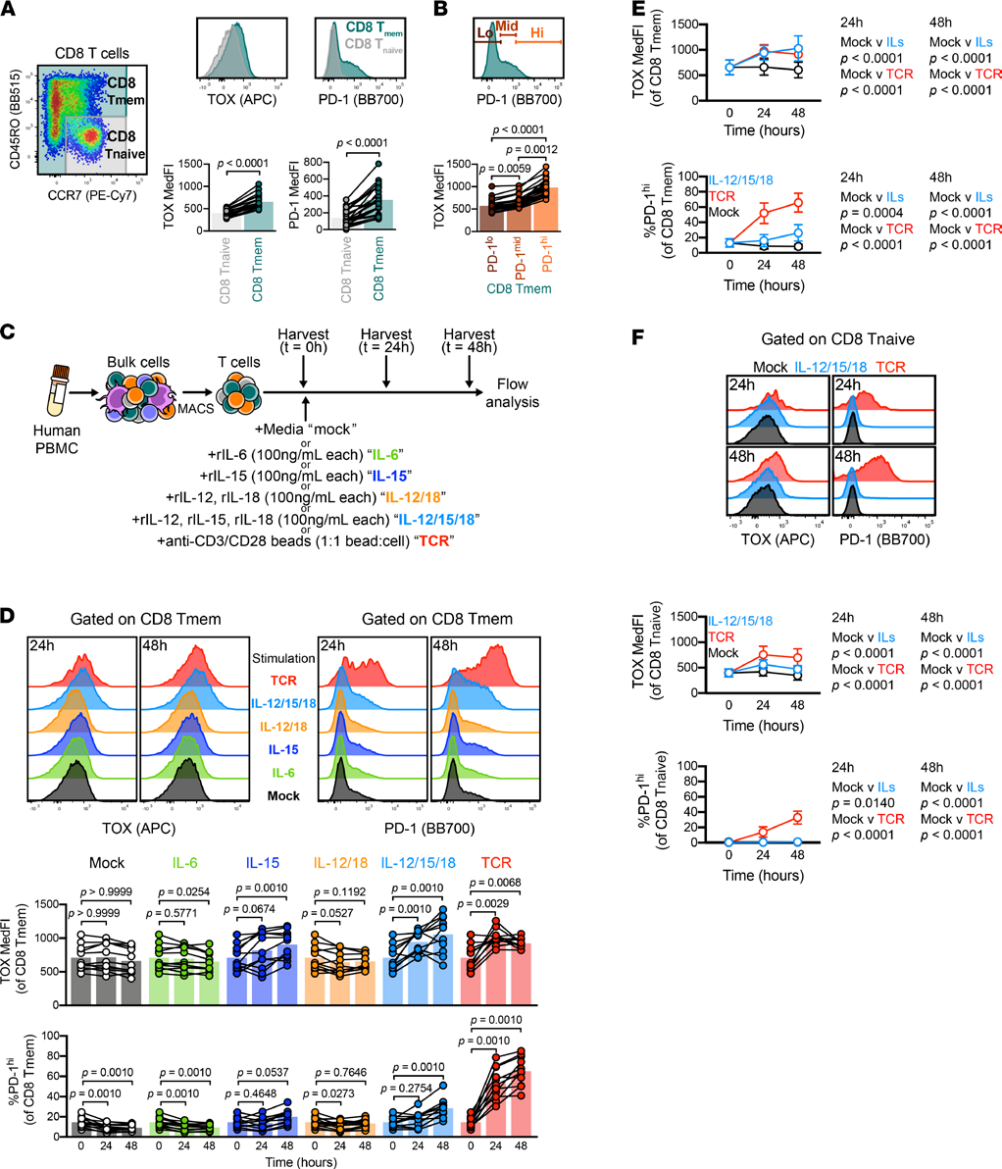
Inflammatory cytokines are potent inducers of TOX and PD-1 in human Tmem cells. (**A**) Basal expression of TOX and PD-1 in CD8^+^ Tmem and Tnaive cells. (**B**) TOX MedFI across PD-1 low–, medium–, and high–expressing CD8^+^ Tmem cells. (**C**) Schematic detailing T cell isolation from cryopreserved PBMCs and subsequent stimulation with recombinant IL-6, IL-15, IL-12/18, and IL-12/15/18 (all at 100 ng/mL, each), or anti-CD3/CD28 microbeads (TCR, 1:1 bead/cell ratio) and subsequent flow interrogation. (**D**) TOX expression (MedFI) and PD-1^hi^ frequency in CD8^+^ Tmem cells throughout stimulation time course. (**E** and **F**) Comparison of TOX MedFI and PD-1^hi^ frequency in mock-, IL-12/15/18–, and TCR-stimulated (**E**) CD8^+^ Tmem and (**F**) CD8^+^ Tnaive cells. In **A**, **B**, and **D**–**F**, we calculated indicated statistical significances by (**A** and **D**) Wilcoxon’s matched-pairs signed-rank tests, (**B**) Friedman’s test with Dunn’s multiple comparisons tests, or (**E** and **F**) Mann-Whitney *U* tests. In **A** and **D**, each symbol represents a unique time point/treatment connected by donor with bars indicating mean (**A**, *n* = 23 across 4 experiments; **D**, *n* = 11 across 2 experiments). In **E** and **F**, each symbol represents the mean ± SD of the stimulation condition from *n* = 23 donors across 4 experiments. Representative plots from **A**, **D**, and **F** are sourced from the same donor. TOX, thymocyte selection–associated high-mobility group box; Tmem, memory T cells; Tnaive, naive T cells; PD-1, programmed cell death protein 1; MedFI, median fluorescence intensity; TCR, T cell receptor.

Using cryopreserved PBMCs from healthy, HIV-seronegative donors, we interrogated TOX and PD-1 expression by flow cytometry. We specifically gated CD8^+^ T cells by a memory and naive binary, delineating CD8^+^ Tnaive cells as CD45RO^–^CCR7^+^, with remaining cells as CD8^+^ Tmem cells (ref. 39; Figure 4A), and interrogated basal TOX and PD-1 expression between these 2 subsets (Figure 4A). Because PD-1 expression is heterogeneous in humans (40, 41), we measured TOX MedFI across PD-1 low–, medium–, and high–expressing events. We found that CD8^+^ Tmem cells with the highest PD-1 expression also demonstrated significantly elevated TOX MedFI (Figure 4B), mirroring correlations of TOX and PD-1 expression in our mouse model as well as human HCV infections (6). We next tested whether IL-12/15/18 stimulation increases PD-1 and TOX expression in T cell subsets and included mock and TCR stimulation conditions as negative and positive controls, respectively, but we also included stimulations using rIL-6, rIL-15, or rIL-12 and rIL-18 (Figure 4C). We chose these additional conditions because IL-6 activates CD8^+^ Tnaive cells (as evidenced by CD69 upregulation) and to discern individual activating contributions of each cytokine (Supplemental Figure 4A). Across these conditions, IL-12/15/18–mediated and TCR-mediated stimulations led to the most prominent increase of TOX staining intensity and PD-1^hi^ frequency in CD8^+^ Tmem cells (Figure 4, D and E). We measured TCF1 expression after mock, IL-12/15/18, and TCR stimulation. A decrease in TCF1 expression accompanied an increase in TOX and PD-1 expression after IL-12/15/18 or TCR stimulation (Supplemental Figure 4B), akin to our mouse stimulation data. We further tested the degree of similarity between human and mouse T cells by measuring PD-1, TCF1, and TOX expression profiles in stimulated human CD8^+^ Tnaive cells. Like mouse CD8^+^ Tnaive cells, only TCR stimulation led to appreciable changes in TOX and PD-1 within human CD8^+^ Tnaive cells (Figure 4F and Supplemental Figure 4C). Because IL-6 can activate CD8^+^ Tnaive cells, we used this condition to determine if PD-1 and TOX expression could occur in Tnaive cells in the absence of a TCR signal. Despite inducing CD69 expression, we found that IL-6–mediated stimulation failed to increase TOX or PD-1 expression in CD8^+^ Tnaive cells (Supplemental Figure 4D). Together, these data show that CD8^+^ Tmem cells differentially expressed TOX, PD-1, and TCF1 at homeostasis and after both IL-12/15/18 and TCR stimulation. We next wanted to better define these changes across different Tmem subsets.

### Inflammation-induced PD-1 and TOX expression occurred in most but not all CD8^+^ Tmem subsets.

**Figure 5:**
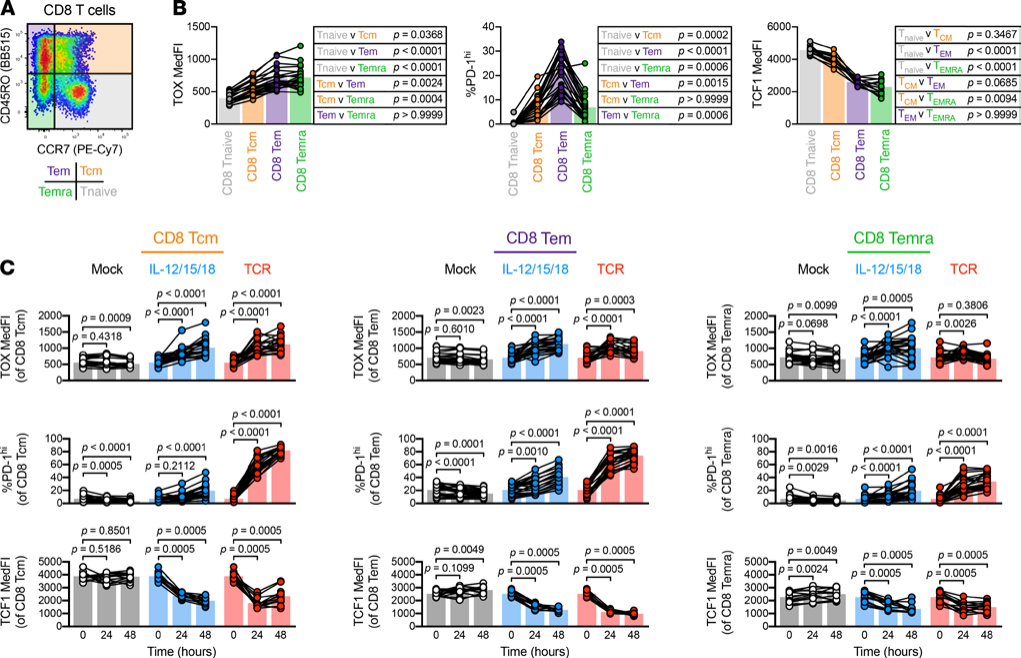
TOX and PD-1 upregulation are largely independent of Tmem subset. Basal and stimulation-induced TOX and PD-1 expression in CD8^+^ memory subsets. (**A**) Representative gating of CD8^+^ T cells into Tnaive (gray), Tcm (orange), Tem (purple), and Temra (green) subsets. (**B**) Basal expression levels (MedFI) of TOX and TCF1 and frequency of PD-1^hi^ cells across CD8^+^ T cell memory subsets. (**C**) TOX MedFI, PD-1^hi^ frequency, and TCF1 MedFI after mock (black), IL-12/15/18 (each at 100 ng/mL, blue), or TCR (1:1 bead/cell ratio, red) stimulation in CD8^+^ Tcm (left column), CD8^+^ Tem (center column), and CD8^+^ Temra (right column) cells. Symbols in **B** and **C** represent unique samples (by time point/condition/subset) and are connected by donor identity, with bars representing mean. We determined statistical significances in **B** and **C**, respectively, using Friedman’s tests and Wilcoxon’s matched-pairs signed-rank tests. **B** and **C** depict *n* = 23 donors across 4 experiments, except for TCF1 plots, which depict *n* = 12 donors across 2 experiments. TOX, thymocyte selection–associated high-mobility group box; Tmem, memory T cells; Tnaive, naive T cells; PD-1, programmed cell death protein 1; MedFI, median fluorescence intensity; Tem, effector memory T cells; TCR, T cell receptor; Temra, CD45RA–expressing effector memory T cells; Tcm, central memory T cells.

To test if inflammation-induced PD-1 and TOX expression differs across human CD8^+^ Tmem subsets, we used CD45RO and CCR7 staining to further delineate central memory (Tcm; CD45RO^+^ CCR7^+^), Tem (CD45RO^+^ CCR7^–^), and Temra (CD45RO^–^ CCR7^–^) subsets (refs. 39, 42; Figure 5A). When we measured TOX, PD-1, and TCF1 expression across these subsets, we noted that a substantial fraction of CD8^+^ Tem events were PD-1^hi^, and both CD8^+^ Tem and Temra cells expressed elevated levels and lower levels of TOX and TCF1, respectively, at homeostasis (Figure 5B). Although this observation is in line with the initial report demonstrating TOX heterogeneity in human CD8^+^ Tmem subsets (17), it remained unknown if these CD8^+^ Tmem subsets are equally capable of further TOX upregulation after stimulation. We observed that TOX, PD-1, and TCF1 expression kinetics in CD8^+^ Tcm and Tem cells largely resembled one another, with both IL-12/15/18 and TCR stimulation having increased the frequency of PD-1^hi^ events and TOX MedFI but having decreased TCF1 MedFI (Figure 5C). It is worth noting that although TCF1 MedFI in CD8^+^ Tcm cells dropped profoundly after IL-12/15/18 or TCR stimulation, the loss in frequency of TCF1-expressing cells (as defined by subjective gating) was not as pronounced as what we observed in CD8^+^ Tem cells (Supplemental Figure 5A). Although both IL-12/15/18–mediated and TCR-mediated stimulation were able to significantly increase the frequency of PD-1^hi^ events and lower TCF1 MedFI in CD8^+^ Temra cells, the degree of these changes was less pronounced than in CD8^+^ Tcm or Tem cells (Figure 5C). Moreover, CD8^+^ Temra cells did not significantly upregulate TOX expression after TCR stimulation. This, however, was not due to an inability to be stimulated because CD8^+^ Temra cells readily expressed the activation marker CD69 after cytokine-mediated or TCR-mediated stimulation (Supplemental Figure 5A). Finally, it is worth noting that when stimulated with IL-15 alone, CD8^+^ Tcm cells, unlike CD8^+^ Tem and Temra cells, failed to significantly express PD-1 (Supplemental Figure 5B).

**Figure 6:**
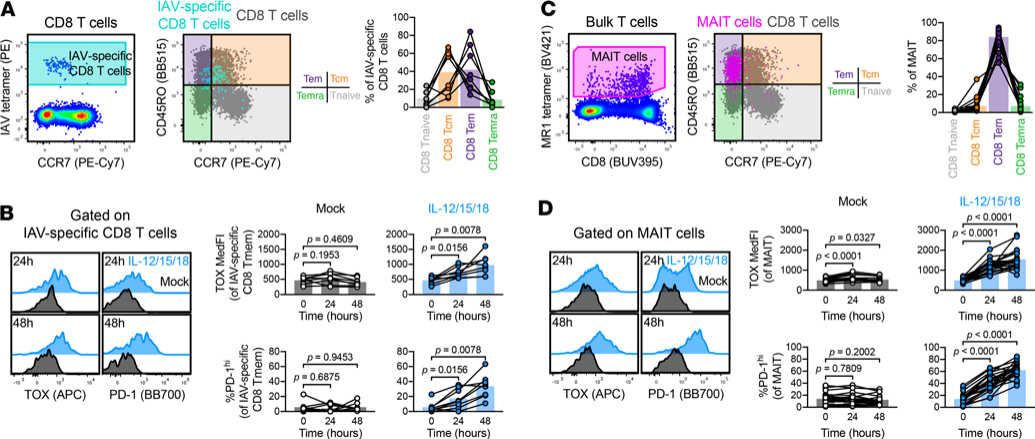
Stimulation induces TOX and PD-1 expression in conventional and innate-like T cells. TOX and PD-1 induction in IAV-specific CD8^+^ T cells. (**A**) Gating and memory phenotyping of IAV-specific CD8^+^ T cells. (**B**) Induction of TOX and PD-1 in IAV- specific CD8^+^ T cells by mock (black) or IL-12/15/18 (each at 100 ng/mL, blue) stimulation. (**C** and **D**) TOX and PD-1 induction in MAIT cells. (**C**) Gating and memory phenotyping of MAIT cells. (**D**) Induction of TOX and PD-1 in MAIT cells by mock (black) or IL-12/15/18 (each at 100 ng/mL, blue) stimulation. Representative plots are sourced from the same donor. Symbols represent unique samples (by time point/condition/subset) and are connected by donor identity, with bars representing mean. We determined statistical significances in **B** and **D** using Wilcoxon’s matched-pairs signed-rank tests. **A** and **B** depict *n* = 8 donors across 2 experiments; **C** and **D** depict *n* = 23 donors across 4 experiments. TOX, thymocyte selection–associated high-mobility group box; PD-1, programmed cell death protein 1; IAV, influenza A virus; MAIT, mucosal associated invariant T.

We next interrogated T cells with defined TCR specificity, specifically IAV-specific CD8^+^ T cells using HLA-A*02 tetramers loaded with the GILGFVFTL peptide (Figure 6A). We examined this CD8^+^ Tmem population because these cells were reported to not express appreciable levels of TOX at homeostasis, likely owing to their Tcm phenotype (17). Within our sample set, IAV-specific CD8^+^ T cells were predominantly Tcm in half of the HLA-A*02 PBMC donors (Figure 6A). Nevertheless, all IAV-specific CD8^+^ T cells were able to substantially upregulate TOX and PD-1 expression after IL-12/15/18 stimulation (Figure 6B), indicating that CD8^+^ Tmem cells with low levels of TOX and PD-1 at homeostasis can also contribute to TOX and PD-1 heterogeneity after recent activation. Alongside testing IAV-specific CD8^+^ T cells, we also interrogated the effects of stimulation in mucosal associated invariant T (MAIT) cells. We selected this population because (a) MAIT cells are nonconventional T cells, recognizing bacterial metabolites as Ags presented on MHC-related 1 (MR1; ref. 43); (b) inflammation is necessary for sustained MAIT cell effector function (44, 45); and (c) MAIT cells are near-uniformly Tem cells when defined by CD45RO and CCR7 (46). We identified MAIT cells using MR1 tetramers loaded with the 5-OP-RU metabolite (47), which largely fell into our Tem gate (Figure 6C). Like IAV-specific CD8^+^ T cells, IL-12/15/18 stimulation led to substantial TOX and PD-1 upregulation in MAIT cells (Figure 6D). Because inflammation is necessary for sustained MAIT cell effector function, we asked if MAIT cells are differentially capable of responding to other cytokine combinations. Alongside IL-12/15/18, IL-15 alone or IL-12 and IL-18 in unison could significantly increase both the frequency of PD-1^hi^ and TOX MedFI of MAIT cells, but not IAV-specific T cells (Supplemental Figure 6, A and B). Together, these data indicate that cytokine-driven activation programs were conserved across conventional and innate-like T cells.

### Cytokine stimulation-induced PD-1 expression was independent of TOX.

**Figure 7:**
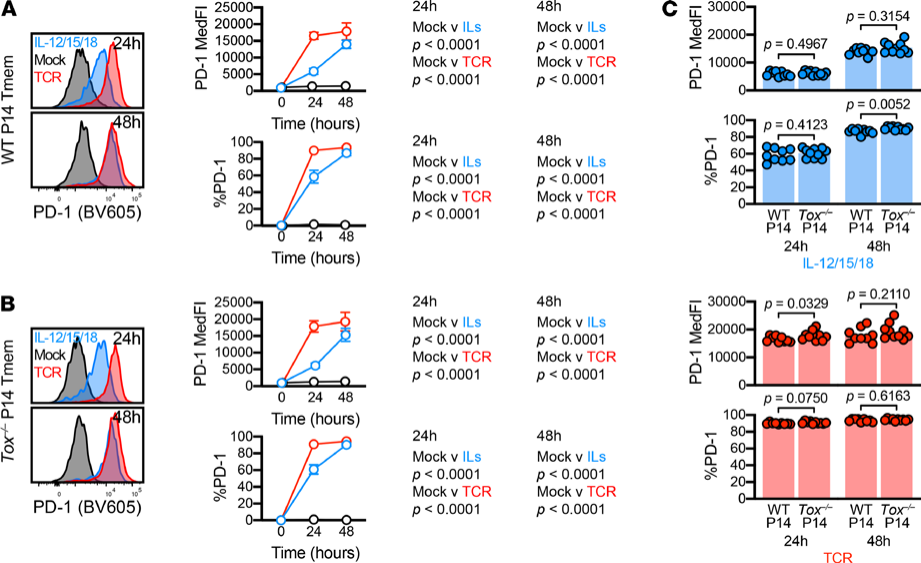
TOX deficiency does not abrogate stimulation-induced PD-1 expression. (Stimulation-induced PD-1 expression in WT and *Tox^–/–^* P14 Tmem cells. T cells were stimulated with media alone (mock), recombinant IL-12, IL-15, and IL-18 in combination (IL-12/15/18 or ILs; each at 100 ng/mL), or with anti-CD3/CD28 microbeads at an approximately 1:1 cell/bead ratio (TCR). (**A** and **B**) PD-1 MedFI and expression frequencies in **A** WT or **B** *Tox^–/–^* P14 Tmem over stimulation time course. (**C**) Comparison of PD-1 MedFI and expression frequencies between IL-12/15/18 (left) or TCR (right) stimulated WT and *Tox^–/–^* P14 Tmem cells. All indicated statistical significances were calculated using Mann-Whitney *U* tests. Symbols in **A** and **B** represent the mean ± SD from all animals at a specific time/condition; and symbols in **C** represent stimulated P14 Tmem populations within a single animal (*n* = 9 WT P14 recipients and *n* = 10 *Tox^–/–^* P14 recipient across 2 experiments). TOX, thymocyte selection–associated high-mobility group box; Tmem, memory T cells; PD-1, programmed cell death protein 1; MedFI, median fluorescence intensity; TCR, T cell receptor.

Finally, because PD-1 and TOX upregulation appeared tightly associated after cytokine-driven activation, we next asked if this association is mechanistic in nature. If TOX is necessary for PD-1 expression, then it would allow the use of surface-expressed PD-1 as a surrogate for intracellularly expressed TOX. TOX expression appears to drive PD-1 expression in a number of contexts because exhausted Tmem cells dramatically downregulate PD-1 after TOX deletion or knockdown (6, 13, 15, 48). Conversely, T cell transduction with TOX-encoding constructs leads to PD-1 upregulation (13–15, 48). Although TOX controls PD-1 expression during exhaustion, the role of TOX is less clear in activation. To dissect the function of TOX in stimulation-mediated PD-1 upregulation, we used WT and *Tox^–/–^* P14 Tmem cells. To generate these P14 Tmem cells, we adoptively transferred WT or KO P14 T cells into C57BL/6J hosts, which we subsequently infected with LCMV Armstrong to form a Tmem population (Supplemental Figure 7A). To determine if TOX deficiency alters stimulation-induced PD-1 upregulation, we cultured MACS-isolated T cells from WT and *Tox^–/–^* P14 memory mice (28 days after LCMV Armstrong infection) in the presence of mock, IL-12/15/18, or TCR stimulation (Supplemental Figure 7A). Both WT and *Tox^–/–^* P14 Tmem cells increased PD-1 expression after IL-12/15/18 or TCR stimulation (Figure 7, A–C). Together these data indicate that TOX alone was not necessary for PD-1 upregulation in cytokine-stimulated CD8^+^ Tmem cells and suggest other transcription factors were sufficient to drive PD-1 expression in the absence of TOX.

## Discussion

TOX has been foremost studied in TCR-mediated exhaustion of mouse CD8^+^ T cells in the context of tumor or chronic infection (6, 13, 14). A recent study reported TOX expression in functional circulating human CD8^+^ Tmem cells, suggesting TOX expression does not necessarily dictate dysfunction (17, 18), which led to the speculation that TOX may have distinct roles across species, specifically mice and humans (19). Alternatively, TOX expression heterogeneity in humans may simply reflect the more complex environment that human T cells are exposed to in everyday life, which may not be readily appreciable in specific pathogen–free mice, such as routine inflammatory events in barrier tissues. Thus, we asked if proinflammatory cues could be sufficient to increase TOX expression and contribute to TOX heterogeneity. Although inflammation has been previously shown to enhance TCR-mediated TOX upregulation (in a VEGF-A–dependent manner that necessitates initial TCR signaling; ref. 49), our findings are, to the best of our knowledge, the first to demonstrate TOX expression in the absence of agonist TCR signals. Transient IL-12/15/18 and TCR stimulation increased PD-1 and TOX expression in most CD8^+^ Tmem cells. In mouse, dysfunctional P14 Tmem cells from LCMV Docile–infected mice still increased surface PD-1 expression after TCR stimulation, whereas IL-12/15/18 had little to no effect on TOX expression. Similarly, human Temra cells showed limited to no increase in TOX expression after exposure to IL-12/15/18. The underlying mechanisms will require further investigation, but one could speculate that the cytokine stimulation was simply not potent enough to further enhance the already ongoing effector or activation program in these 2 Tmem subsets. The notion that not only TOX but also PD-1 expression can indicate an ongoing effector or activation program in CD8^+^ T cells is important because PD-1 and (now also) TOX are used as biomarkers of T cell exhaustion (50–52). Of note, certain features of general activation programs of CD8^+^ Tmem cells appear to be well conserved and have also been reported as transcriptomic overlap of tissue-resident, recently activated, and exhausted CD8^+^ T cells (53). Although infection parameters and inflammatory events are well defined in mouse model studies, most human studies remain agnostic in regard to the infection and activation history of Ag-specific T cells. This in turn makes it difficult to correctly interpret the underlying reason for expression of PD-1 and TOX by human T cells.

Our data emphasize the need for conservative interpretation of TOX in regard to activation and exhaustion and also caution against interpreting TOX expression purely through the lens of recent TCR-mediated activation. TOX expression is predictive of T cell exhaustion and unfavorable outcome in hepatocellular carcinoma animal models and clinical samples (54), in line with the paradigm of TOX-mediated, TCR-dependent T cell dysfunction. However, other studies have yielded contradictory data. Meta analyses of TOX expression in breast cancers reported TOX levels paradoxically correlating with increased immune cell function and favorable prognosis (55). This is perplexing, as in tumors, TOX expression is associated with T cell dysfunction (6, 13, 14). This discrepancy could be in part explained by TOX upregulation during activation, akin to what we observed during T cell activation in TCR-dependent and -independent stimulations. Thus, our data stress that all possible activation pathways of TOX and PD-1 induction must be considered before interpreting TOX as a biomarker of T cell dysfunction. A well-done human study (17) that interrogated TOX heterogeneity found elevated TOX in CMV-specific and EBV-specific CD8^+^ Tmem cells and hypothesized that recent viral reactivation provide cognate Ag to facilitate TCR-mediated upregulation of TOX. This is certainly a plausible explanation, but our data highlight the need to also consider recent exposure to inflammation as a critical parameter affecting TOX expression. Conventional CD8^+^ Tem and Temra cells (the predominant phenotype of CMV-specific and EBV-specific CD8^+^ T cells) express elevated levels of TOX basally; Tcm (including IAV-specific CD8^+^ T cells) and innate-like MAIT cells can, too, upregulate TOX expression after inflammation-mediated activation. Importantly, our data highlight that this mechanism of TOX expression was conserved across species, conventional CD8^+^ Tmem subsets, and innate-like MAIT cells.

Because proinflammatory cytokines can concurrently induce TOX and PD-1 expression, these signals may drive TOX heterogeneity in other contexts. P14 tissue-resident Tmem cells show increased *Tox* expression at homeostasis, which is observed 90 days after priming with LCMV Armstrong (20). Because the acute infection is cleared well before this time point, it is unlikely that continued TCR signaling by cognate Ag drives this phenotype, despite elevated transcripts encoding mediators of TCR signaling (20). However, IL-15 is likely present within the tissue microenvironment. IL-15 is implicated in Trm cell maintenance (25, 26), and transcriptional profiles indicative of IL-15/STAT5 signaling are detected in human Trm (23, 24). Thus, IL-15 in tissue microenvironments may also contribute to TOX heterogeneity. Future work will be necessary to dissect the role of these inflammatory cues versus other signals that can shape Trm phenotype, such as costimulation and tonic TCR signaling (21).

Previous studies have demonstrated that TOX ablation or knockdown leads to PD-1 downregulation in models of exhaustion (6, 13, 15, 48), and, conversely, introduction of TOX-expressing constructs enhances PD-1 expression (15, 48). Similarly, our data showed a close correlation in regards to TOX and PD-1 expression levels, but we found that PD-1 expression could be induced in stimulated *Tox^–/–^* P14 Tmem cells. Of note, these *Tox^–/–^* P14 Tmem cells lacked exon 5, which abrogates the ability to function as a transcription factor, but the truncated protein is still expressed and detected by the TOX antibody. Alfei et al. (6) previously showed that the early wave of effector cells formed from *Tox^–/–^* Tnaive cells express significant levels of PD-1 independently of functional TOX. However, TOX is required for the expression of high levels of PD-1 at later stages, once the initial population of exhausted effector T cells are replaced by a proliferation competent TCF1 progenitor population (31). Together, these data suggest that the long-term expression of PD-1 requires TOX, but the activation-induced expression of PD-1 is TOX-independent. In the absence of TOX, PD-1 expression could be driven by TOX2, which can induce PD-1 expression in CD8^+^ T cells (15, 48); however, it remains unclear if TOX2 is also upregulated by transient TCR-mediated or cytokine-mediated stimulation. Similarly, how different activating signals integrate to regulate TOX expression also requires further studies: although inflammatory cues increase TOX expression in Tmem cells, increased IL-12 signaling during the priming of Tnaive cells has been shown to limit subsequent TOX expression at steady state (56, 57).

Overall, our data suggest that the mechanisms that regulate TOX expression, both at homeostasis and after transient TCR or cytokine stimulation, were remarkably similar and quite possibly highly conserved between humans and mice. Our data further highlight the need to consider TOX and PD-1 expression as prominent indicators of ongoing activation and effector programs in Tmem instead of exclusive biomarkers of exhaustion.

## Methods

### Mice.

All animals were maintained in specific pathogen–free facilities and infected in modified pathogen–free facilities. Experimental groups were nonblinded; animals were randomly assigned to experimental groups; and no specific method was used to calculate sample sizes.

We purchased 6-week-old female C67BL/6J mice from The Jackson Laboratory; *Tox^–/–^* P14 mice (P14 *Tox^tm1c(KOMP)Wtsi^*;*Mx^Cre^*;*Rosa26-STOP-eYFP*) were generated as previously described (6). WT and *Tox^–/–^* P14 mice, OT-I mice, and gBT-I mice were maintained on CD45.1 congenic backgrounds. We euthanized mice in accordance with institutional protocols and subsequently collected spleens and LNs for experimentation.

### Development of memory mice.

We prepared a single-cell suspension of LN cells that were harvested from female OT-I, P14, or gBT-I mice by mechanically passing LN tissue through a 70 to 100 μm strainer. To enrich transgenic T cells, we used MACS with a CD8 Negative Selection Kit (Miltenyi Biotec).

For OT-I memory mice, we adoptively transferred 1 × 10^4^ OT-I T cells in sterile 1 × PBS i.v. per C57BL/6J recipient and subsequently infected recipients i.v. with 1 to 2 × 10^7^ PFU VSV-OVA or 4 × 10^3^ CFU OVA-expressing *L*. *monocytogenes* (*L*. *monocytogenes*–OVA). For gBT-I memory mice, we adoptively transferred 5 × 10^4^ gBT-I T cells i.v. and subsequently infected recipient mice i.v. with or 4 × 10^3^ CFU HSV2 gB–expressing *L*. *monocytogenes* (*L*. *monocytogenes*–gB). We allowed 60 days or longer to pass after initial VSV or *L*. *monocytogenes* infections before assaying tissues.

For P14 memory mice, we adoptively transferred 2 × 10^3^ WT P14 T cells i.v. and subsequently infected recipient mice i.v. with 2 × 10^5^ PFU LCMV Armstrong clone (LCMV Arm.) or 2 × 10^6^ PFU LCMV Docile clone (LCMV Doc.). For *Tox^–/–^* P14 memory mice, we adoptively transferred 2 × 10^3^ *Tox^–/–^* P14 memory mice and subsequently infected with 2 × 10^5^ PFU LCMV Arm.; we allowed 28 days to pass after initial LCMV infection before assaying tissues.

### T cell isolation and in vitro stimulation.

We harvested spleen and LN from memory mice and mechanically prepared single-cell suspensions. We thawed approximately 4 × 10^7^ cryopreserved PBMC in human RP10 media (RPMI1640 supplemented with 10% FBS, 2 mM L-glutamine, 100 U/mL penicillin-streptomycin). To enrich bulk T cells from single-cell suspensions, we respectively used mouse-specific and human-specific T cell negative isolation MACS (STEMCELL Technologies). We plated 0.5 to 1 × 10^6^ T cells per well in 96-well V-bottom tissue culture plates. We cultured cells in human RP10 or mouse RP10 media (RPMI 1640 supplemented with 10% FBS, 2 mM L-glutamine, 100 U/mL penicillin-streptomycin, 1 mM sodium pyruvate, 0.05 mM β-mercaptoethanol, and 1 mM HEPES). To stimulate cells, we cultured mouse T cells in mouse RP10 with rIL-12, rIL-15, and rIL-18 (each at 100 ng/mL; BioLegend), with Dynabeads mouse T-Activator (Thermo Fisher) anti-CD3/CD28 beads (at a 1:1 bead/cell ratio) or media alone. For human T cell stimulations, we used human RP10 media with combinations of rIL-6 (BioLegend), rIL-12, rIL-15, and/or rIL-18 (each at 100 ng/mL; Peprotech), with Dynabeads human T-Activator (Thermo Fisher) anti-CD3/CD28 beads (at a 1:1 bead/cell ratio) or RP10 alone. We cultured cells at 37°C, 5% CO_2_, sampling cells at 0, 24, and 48 hours for flow staining. For intracellular cytokine staining, we added GolgiPlug (BD Biosciences) at a 1:1000 dilution 8 hours prior to cell harvest.

### Flow cytometric analysis.

We conducted all flow staining for mouse and human T cells on ice and at room temperature, respectively. All mouse and human flow panel reagent information, stain conditions, and gating are included in Supplemental Figures 8–11 and Supplemental Tables 1–6. We conducted LIVE/DEAD fixable aqua or blue viability dye (Thermo Fisher) (AViD or BViD, respectively) or Zombie Near-IR viability dye (NIRViD) (BioLegend) staining in 1 × PBS. For surface staining, we utilized FACS Wash (1 × PBS supplemented with 2% FBS and 0.2% sodium azide) as the stain diluent. For all TOX staining panels, we fixed cells with the FOXP3 Fixation/Permeabilization Buffer Kit (Thermo Fisher) and conducted intranuclear stains using the FOXP3 Permeabilization Buffer (Thermo Fisher) as diluent. To minimize day-to-day variation for TOX staining, we conducted all intracellular stains within a batch (0-, 24-, and 48-hour samples) at the same time. We resuspended cells in FACS Wash and acquired events on a FACSymphony A5 and LSRFortessa cell analyzers (BD Biosciences), which we analyzed using FlowJo v10 (BD Biosciences). We conducted statistical testing using Prism v8 (GraphPad).

### Statistics.

We used 2-tailed paired *t* tests, Mann-Whitney *U* tests, Wilcoxon’s matched-pairs signed-rank tests, as well as Friedman’s tests with Dunn’s multiple comparisons tests.

### Study approvals.

Mouse protocols and experimentation conducted at the Fred Hutchinson Cancer Research Center were approved by and in compliance with the ethical regulations of the Fred Hutchinson Cancer Research Center’s IACUC. Experiments performed at the Technical University of Munich were in compliance with institutional and governmental regulations in Germany and approved by the veterinarian authorities of the Regierung von Oberbayern in Germany.

Twenty-three healthy, HIV-uninfected adults were recruited by the Seattle HIV Vaccine Trials Unit (Seattle, Washington, USA) as part of the study “Establishing Immunologic Assays for Determining HIV-1 Prevention and Control.” These samples are also known as the Seattle Area Control Cohort. All participants were provided and signed informed consent, and the Fred Hutchinson Cancer Research Center IRB approved the study protocol.

## Author contributions

NJM, JB, DZ, and MP designed research studies. NJM, JB, and AKT conducted the experiments and acquired the data. NJM, JB, AKT, DZ, and MP analyzed the data. NJM, JB, DZ, and MP wrote the manuscript, and NJM, JB, AKT, DZ, and MP edited the manuscript to its final format. NJM. conceived the initial study and was therefore designated the first-listed co–first author for this study.

## Supplementary Material

Supplemental data

## Version 1 05/25/2021

In-Press Preview

## Version 2 07/08/2021

Electronic publication
