# Supplementary material for: Inflammatory signals are sufficient to elicit TOX expression in mouse and human CD8+ T cells
Source: JCI Insight. 2021 Jul 8;6(13):e150744. doi: 10.1172/jci.insight.150744 (PMC8410038; doi:10.1172/jci.insight.150744)
Supplement: Supplemental data [file jciinsight-6-150744-s159.pdf]

Supplemental figures
Maurice and Berner, *et al.*

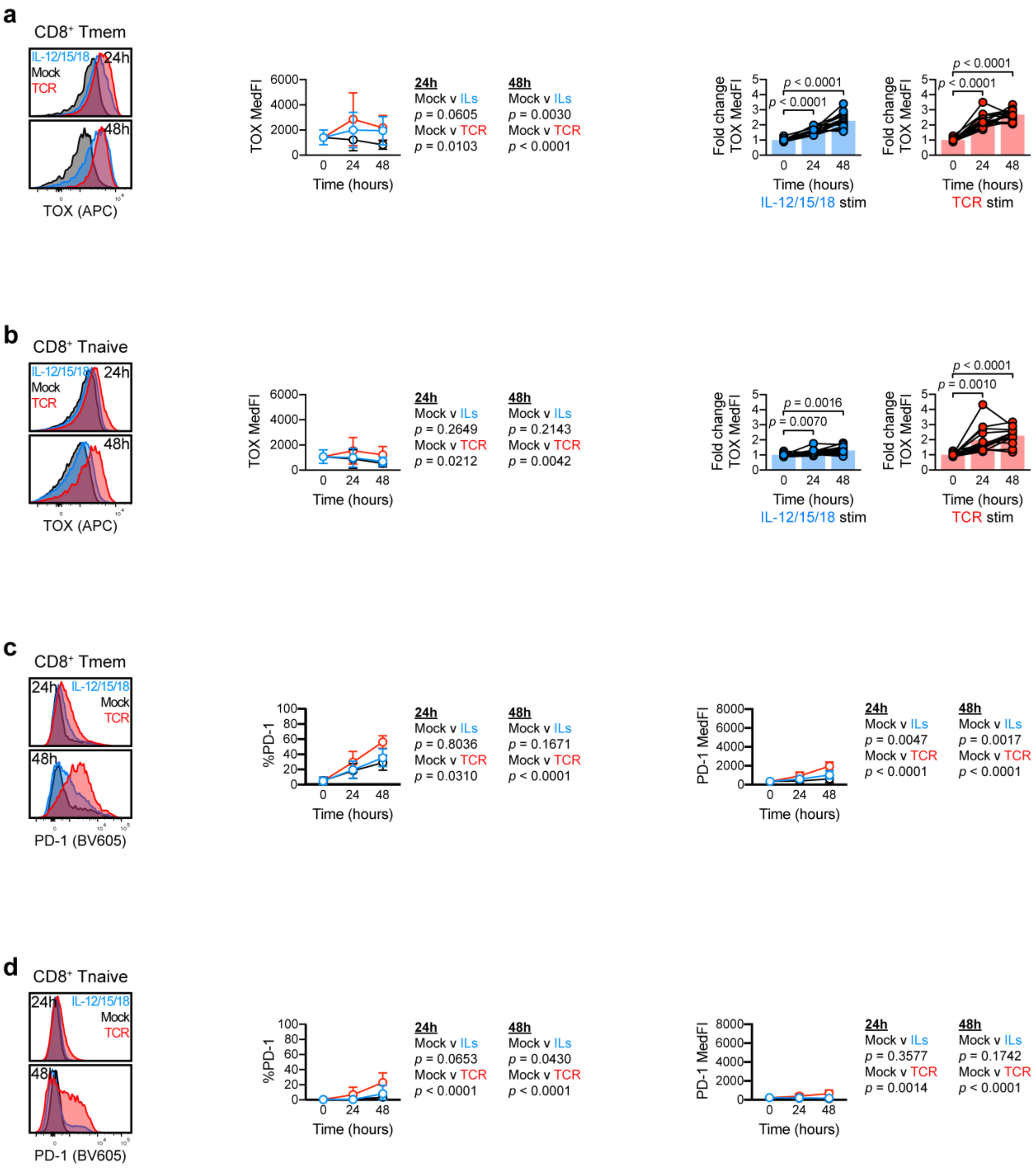

Supplemental Fig. 1, related to Fig. 1: Cytokine stimulation induces TOX expression in murine CD8$^+$ Tmem

a-**d** T cell stimulation responses after culture with media (mock, black), IL-12/15/18 (each at 100ng/mL, blue), or TCR agonist (1:1 bead to cell ratio, red). **a**-**b** Stimulation-induced changes

of TOX expression in **a** endogenous CD8+ Tmem and **b** endogenous CD8+ Tnaive sourced from VSV-OVA OT-I memory mice. **c-d** Stimulation-induced changes of PD-1 expression in **c** endogenous CD8+ Tmem and **d** endogenous CD8+ Tnaive sourced from VSV-OVA OT-I memory mice. TOX MedFI fold changes in **a** and **b** were calculated against average TOX MedFI from mock stimulations in a subset-specific, batch-specific, and timepoint-specific manner. In **a-d**, symbols in line plots comparing stimulation conditions represent the mean across all animals for a specific timepoint/condition ± SD; the indicated statistical significances were calculated using Mann-Whitney tests. In **a** and **b**, bar chart symbols represent one animal at a unique timepoint/condition and are connected by animal identity, with bar indicating mean; the indicated statistical significances were calculated using paired t tests. All figures depict results from *n* = 14 mice across 7 experiments. All representative flow plots are sourced from the same animal.

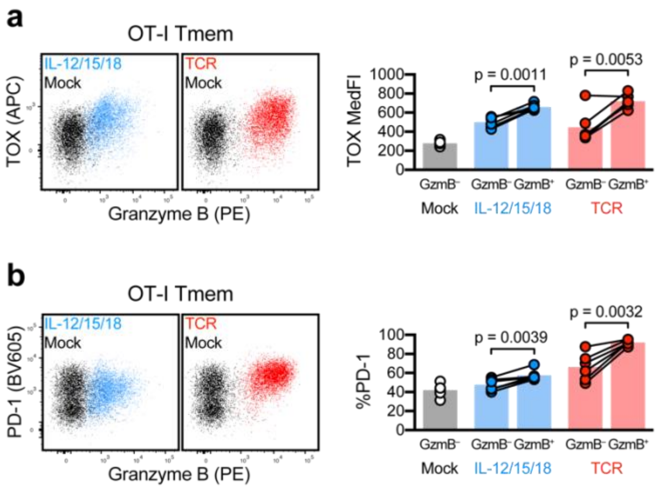

Supplemental Fig. 2, related to Fig. 2: TOX and PD-1 expression occur in functional CD8^{+} T cells

T cells were isolated from VSV-OVA OT-I memory mice and stimulated (mock, black; IL-12/15/18, blue; TCR, red) for 24 hours (schematic shown in **Fig. 2a**). **a-b** Expression of **a** TOX and **b** PD-1 within granzyme B (GzmB)-positive and -negative OT-I Tmem after stimulation. Symbols in **a** and **b** represent a T cell population within a unique animal with symbols connected by animal identity (*n* = 6 across 2 experiments). Bars represent mean and indicated statistical significances were calculated by paired t test.

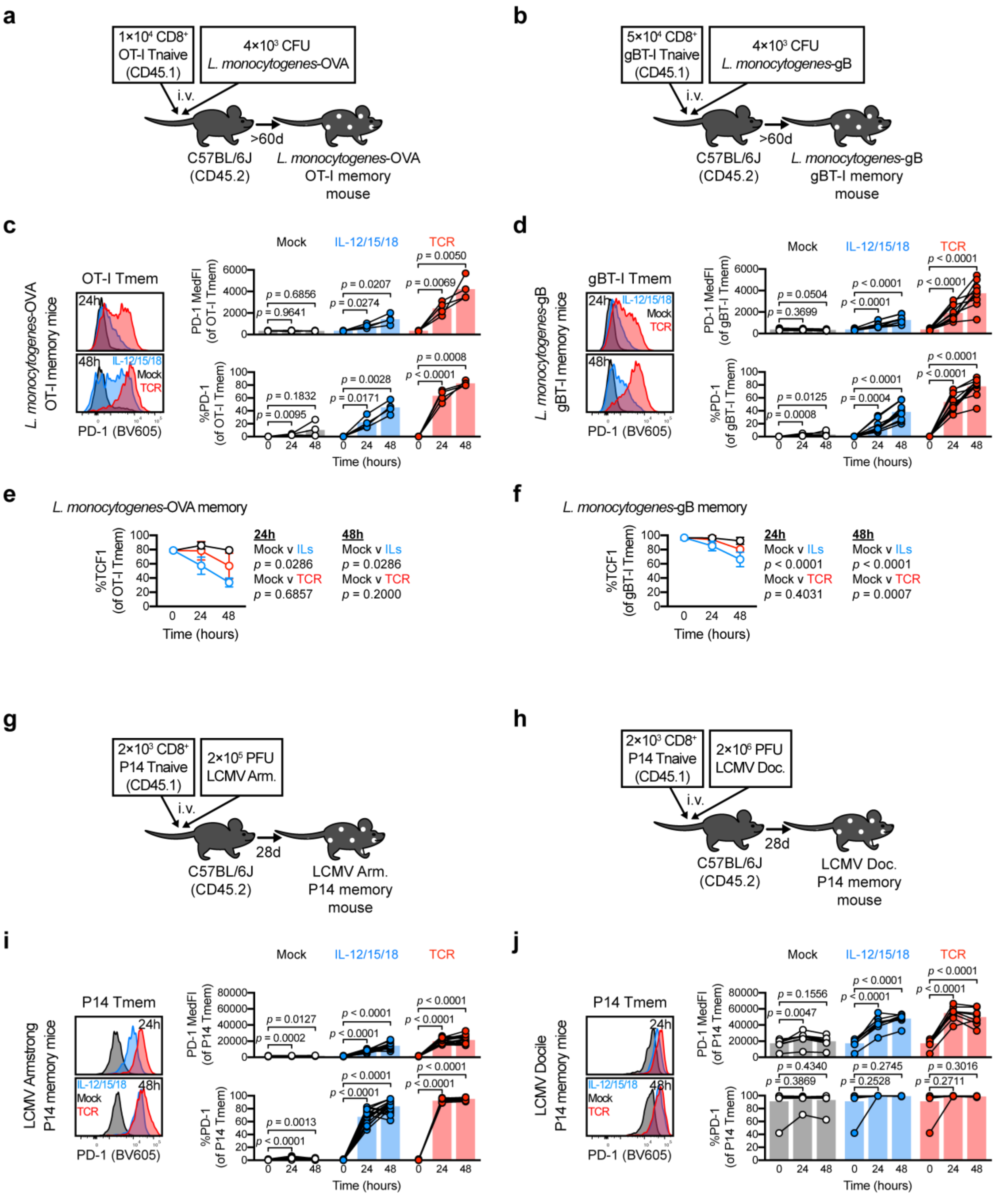

Supplemental Figure 3, related to Fig. 3: TOX induction varies across memory T cells elicited by different infections

a-f Changes in PD-1 and TCF1 expression within *L. monocytogenes*-expanded TCR transgenic Tmem. **a-b** Experiment schematic outlining the expansion of **a** OT-I (OVA-specific) and **b** gBT-I (gB-specific) transgenic T cells with *L. monocytogenes*-OVA and *L. monocytogenes*-gB, respectively. MACS-enriched T cells from these memory mice were stimulated with media alone (mock), recombinant IL-12, -15, and -18 in combination (IL-12/15/18) (each at 100ng/mL), or anti-CD3/CD28 microbeads (TCR) at a ~1:1 cell:bead ratio. **c, d** PD-1 MedFI and expression frequency within stimulated **c** OT-I Tmem or **d** gBT-I Tmem. **e, f** TCF1 expression within stimulated **e** OT-I and **f** gBT-I Tmem. **g-j** Changes in PD-1 expression within LCMV Armstrong- and Docile-expanded P14 Tmem. **g-h** Experiment schematic outlining the expansion of P14 transgenic T cells with **g** LCMV Armstrong (Arm.) or **h** LCMV Docile (Doc.), which respectively cause acute and chronic infection. **i-j** PD-1 MedFI and expression frequency within **i** LCMV Armstrong- and **j** Docile-expanded P14 Tmem. Symbols in **c, d, i, j** represent T cell populations from a single animal at a unique timepoint/condition and are connected by matched donor identities (when applicable), with bars depicting mean. Symbols in **e, f** represent mean values ± SD. Indicated statistical significances in **c, d, i, j** were calculated by paired t tests, and those in **e, f** were calculated using Mann-Whitney tests. Data in **a-f** depict n = 4 *L. monocytogenes*-OVA OT-I memory mice and n = 10 *L. monocytogenes*-gB gBT-I memory mice across 2 experiments. Data in **g** and **j** depict n = 17 LCMV Armstrong expanded P14 memory mice across 4 experiments and n = 8 LCMV Docile expanded P14 memory mice across 2 experiments.

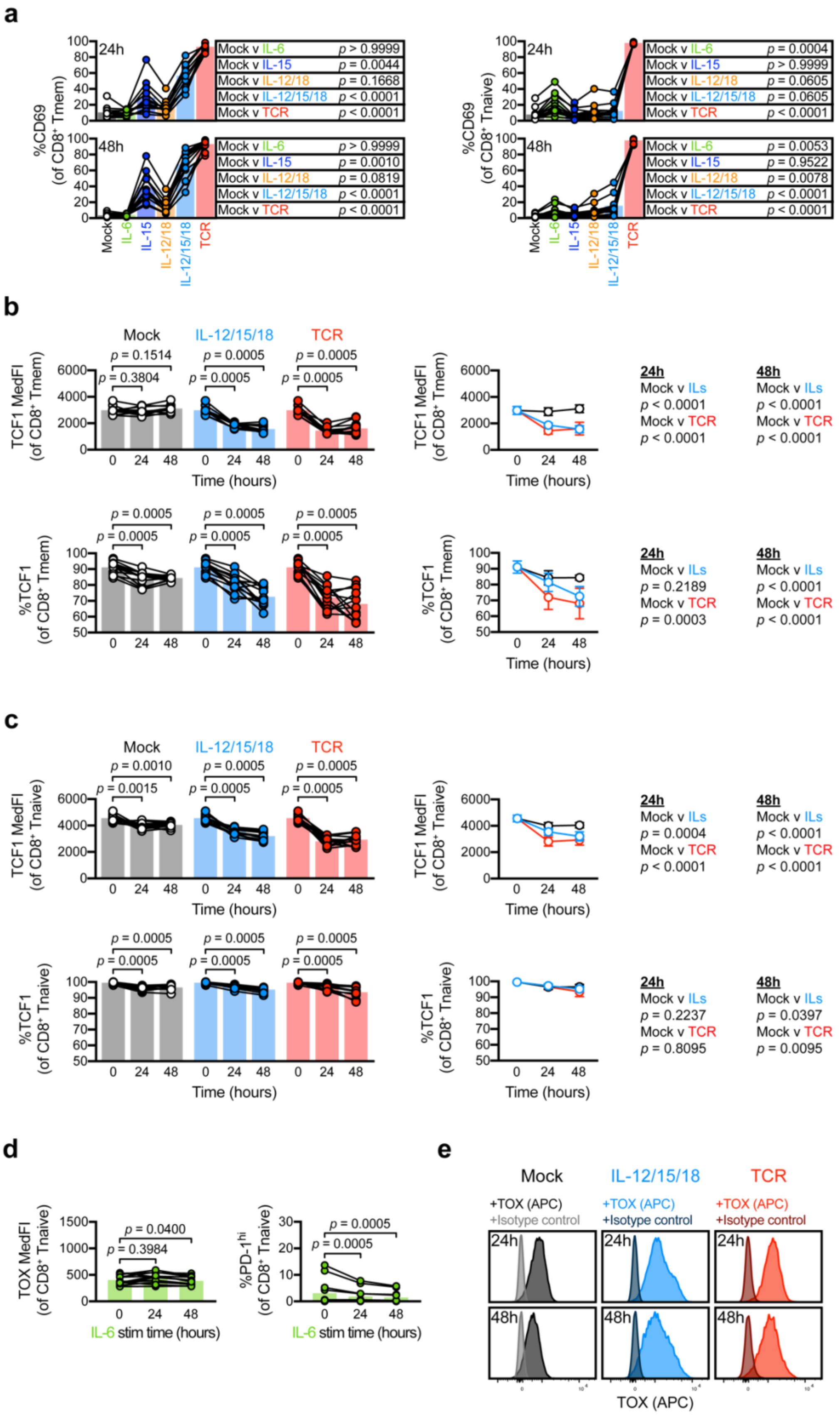
a
%CD69 (of CD8+ Tmem)
24h
Mock v IL-6 p > 0.9999
Mock v IL-15 p = 0.0044
Mock v IL-12/18 p = 0.1668
Mock v IL-12/15/18 p < 0.0001
Mock v TCR p < 0.0001
48h
Mock v IL-6 p > 0.9999
Mock v IL-15 p = 0.0010
Mock v IL-12/18 p = 0.0819
Mock v IL-12/15/18 p < 0.0001
Mock v TCR p < 0.0001
Mock
IL-6
IL-15
IL-12/18
IL-12/15/18
TCR
%CD69 (of CD8+ Tnaive)
24h
Mock v IL-6 p = 0.0004
Mock v IL-15 p > 0.9999
Mock v IL-12/18 p = 0.0605
Mock v IL-12/15/18 p = 0.0605
Mock v TCR p < 0.0001
48h
Mock v IL-6 p = 0.0053
Mock v IL-15 p = 0.9522
Mock v IL-12/18 p = 0.0078
Mock v IL-12/15/18 p < 0.0001
Mock v TCR p < 0.0001
b
Mock
IL-12/15/18
TCR
p = 0.1514
p = 0.3804
p = 0.0005
TCF1 MedFI (of CD8+ Tmem)
Time (hours)
24h
Mock v ILs
p < 0.0001
Mock v TCR
p < 0.0001
48h
Mock v ILs
p < 0.0001
Mock v TCR
p < 0.0001
%TCF1 (of CD8+ Tmem)
24h
Mock v ILs
p = 0.2189
Mock v TCR
p = 0.0003
48h
Mock v ILs
p < 0.0001
Mock v TCR
p < 0.0001
c
Mock
IL-12/15/18
TCR
p = 0.0010
p = 0.0015
p = 0.0005
TCF1 MedFI (of CD8+ Tnaive)
Time (hours)
24h
Mock v ILs
p = 0.0004
Mock v TCR
p < 0.0001
48h
Mock v ILs
p < 0.0001
Mock v TCR
p < 0.0001
%TCF1 (of CD8+ Tnaive)
24h
Mock v ILs
p = 0.2237
Mock v TCR
p = 0.8095
48h
Mock v ILs
p = 0.0397
Mock v TCR
p = 0.0095
d
TOX MedFI (of CD8+ Tnaive)
p = 0.0400
p = 0.3984
IL-6 stim time (hours)
%PD-1hi (of CD8+ Tnaive)
p = 0.0005
p = 0.0005
IL-6 stim time (hours)
e
Mock
IL-12/15/18
TCR
+TOX (APC)
+Isotype control
24h
48h
TOX (APC)

Supplemental Figure 4., related to Fig. 4: Inflammatory cytokines are potent inducers of TOX and PD-1 in human Tmem

a-**b** CD69 expression in stimulated $CD8^+$ Tmem (left) and $CD8^+$ Tnaive (right) subsets. **b**-**c** TCF1 MedFI and expression frequency in **b** $CD8^+$ Tmem and **c** $CD8^+$ Tnaive over stimulation time course (mock, black; IL-12/15/18, blue; TCR, red). **d** TOX MedFI and PD-1^{hi} event frequencies in IL-6-stimulated $CD8^+$ Tnaive. **e** TOX and isotype control staining in mock (left), IL-12/15/18 (center), and TCR (right) stimulated $CD8^+$ Tmem. Bar plot symbols in **a**-**d** depict a unique donor at a specific condition/timepoint, with symbols connected by donor identity and bars depicting mean; indicated statistical significances were calculated by **a** Friedman tests with Dunn's multiple comparisons tests or **b**-**d** Wilcoxon matched-pairs signed rank tests. Line plot symbols in **b** and **c** depict means ± SD across stimulation conditions with indicates statistical significances calculated by Mann-Whitney tests. **a** and **d** represent n = 11 donors across 2 experiments; **b** and **c** represent n = 12 donors across 2 experiments; **e** represents n = 3 donors.

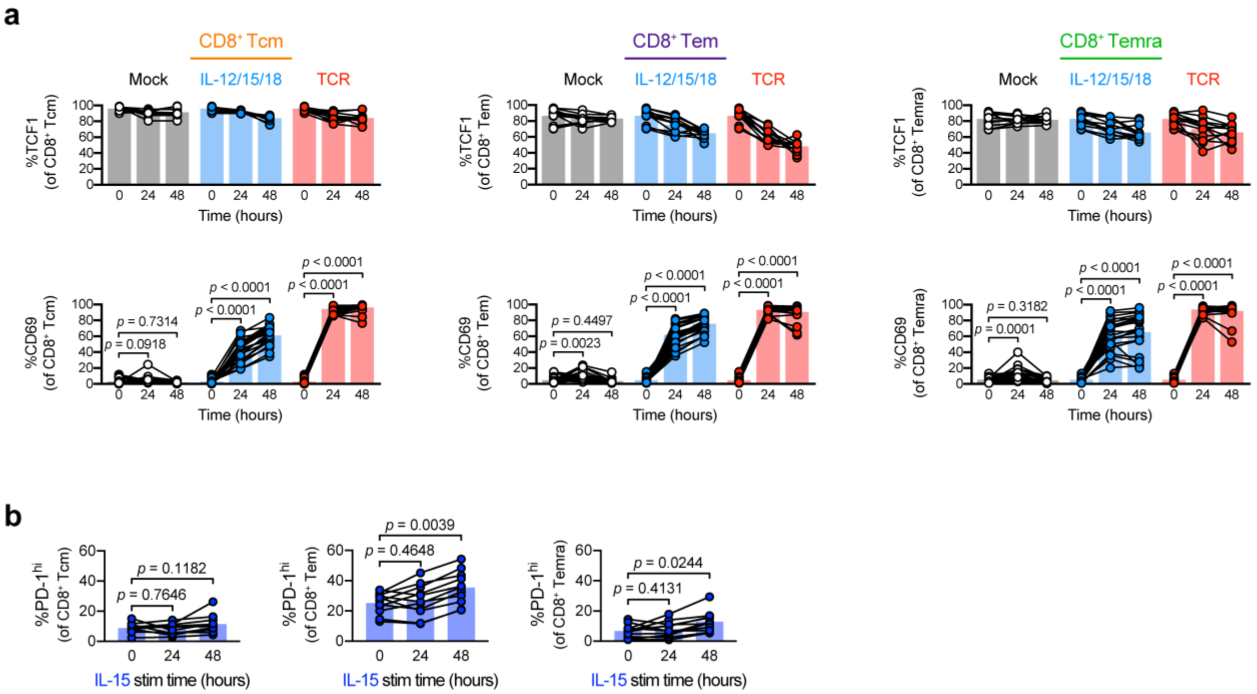

Supplemental Figure 5, related to Fig. 5: TOX and PD-1 upregulation are largely independent of Tmem subset

a TCF1 (top row) and CD69 (bottom row) event frequency within CD8$^+$ Tmem subsets after mock (black), IL-12/15/18 (blue), or TCR (red) stimulation. **b** PD-1^{hi} event frequency within CD8$^+$ Tmem subsets after IL-15 stimulation. All cytokines were at 100ng/mL, each, in all stimulation conditions. In **a**-**b**, each symbol represents a cell population within a donor at a unique condition/timepoint, with symbols connected by donor identity and bar representing mean. All indicated statistical significances were calculated by Wilcoxon matched-pairs signed rank tests. Data in **a** depicts 12 (%TCF1) donors over 2 experiments and 23 donors (%CD69) over 4 experiments; **b** depicts 11 donors over 2 experiments.

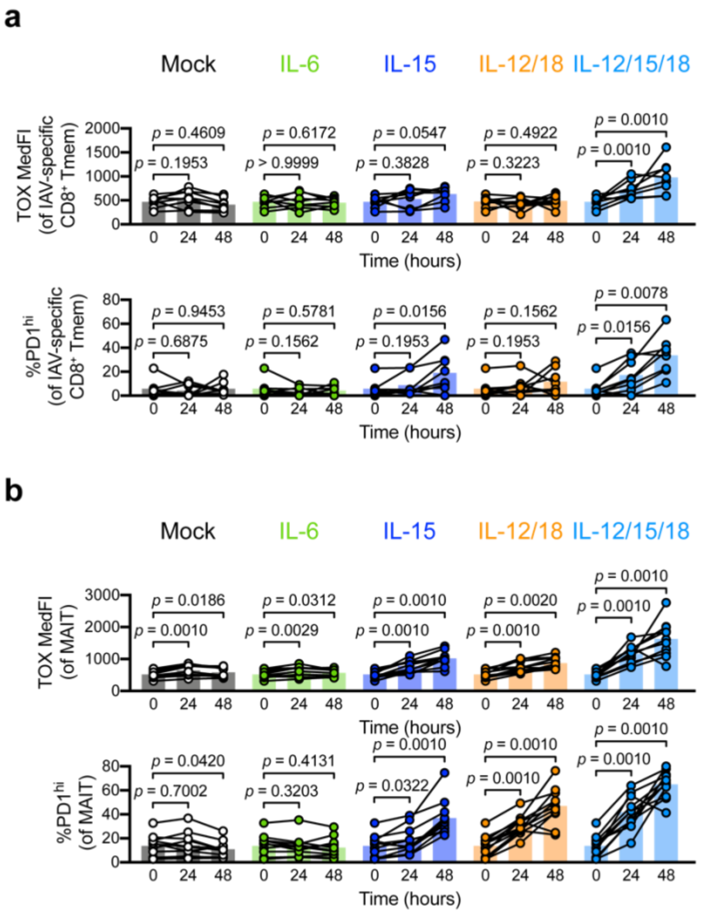

Supplemental Figure 6, related to Fig. 6: Stimulation induces TOX and PD-1 expression in conventional and innate-like T cells

a-**b** TOX MedFI and frequency of PD-1^{hi} events in **c** IAV-specific CD8^{+} T cells and **d** MAIT cells after mock (black), IL-6 (green), IL-15 (dark blue), IL-12/18 (orange), or IL-12/15/18 (blue) stimulation (all cytokine concentrations 100/ng/mL, each). Data in **a** depicts 8 donors over 2 experiments; **b** depicts 11 donors over 2 experiments. All indicated statistical significances were calculated using Wilcoxon matched-pairs signed rank tests.

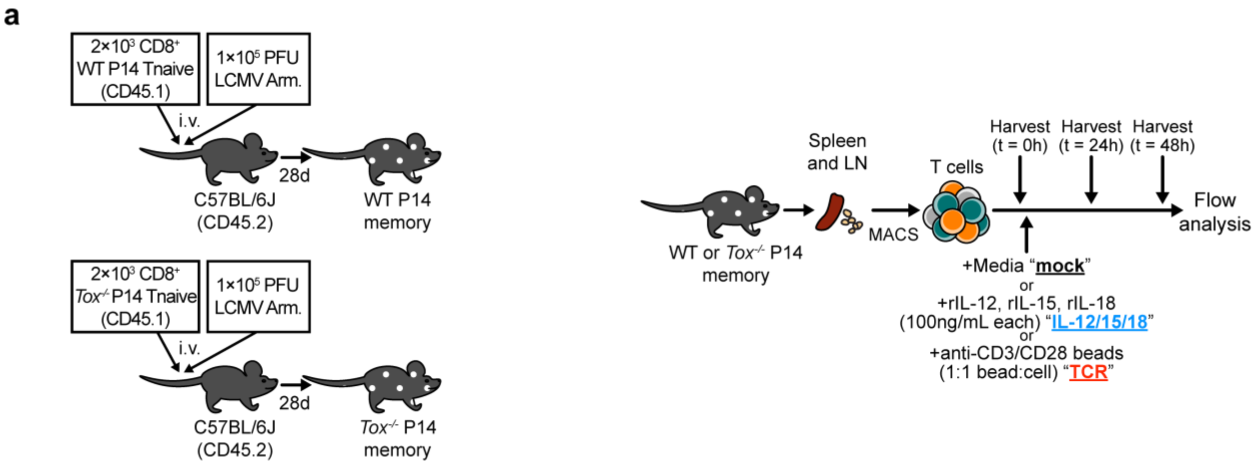

Supplemental Figure 7, related to Fig. 7: TOX deficiency does not abrogate stimulation-induced PD-1 expression

a Experiment schematic for adoptive transfers, tissue harvests, and stimulations conducted in Fig. 7.

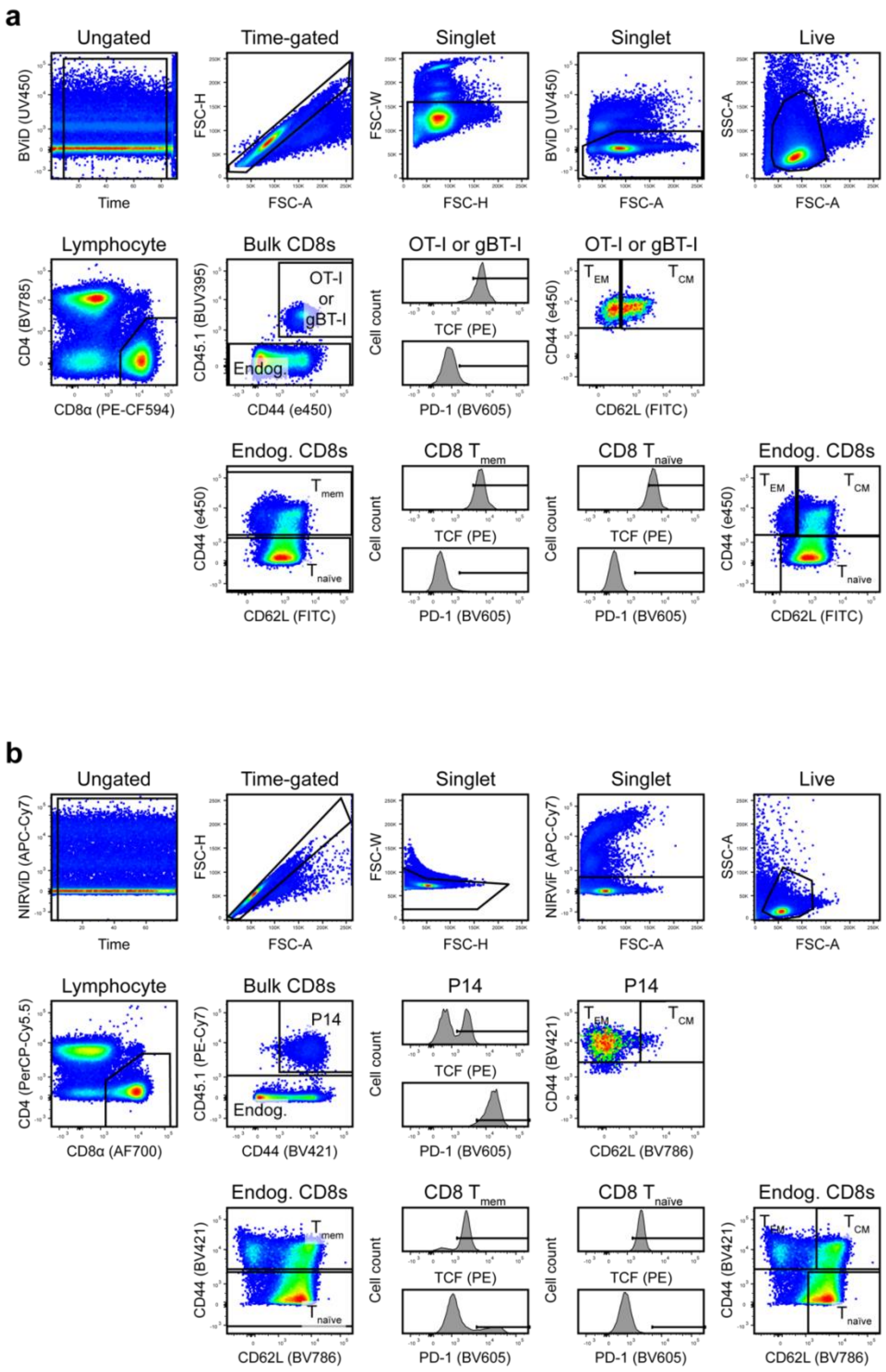

Supplemental Figure 8, related to Fig. 1, 3, 7, Supplemental Fig. 1, 3

a-**b** Representative flow gating for **a** VSV-OVA OT-I memory mice, LM-OVA OT-I memory mice, and LM-gB gBT-I memory mice (unstimulated, 0 h timepoint, VSV-OVA OT-I memory mouse) and **b** LCMV Armstrong- and Docile-expanded WT and Tox–/– P14 memory mice (unstimulated, 0 h timepoint, LCMV Docile WT P14 memory mouse).

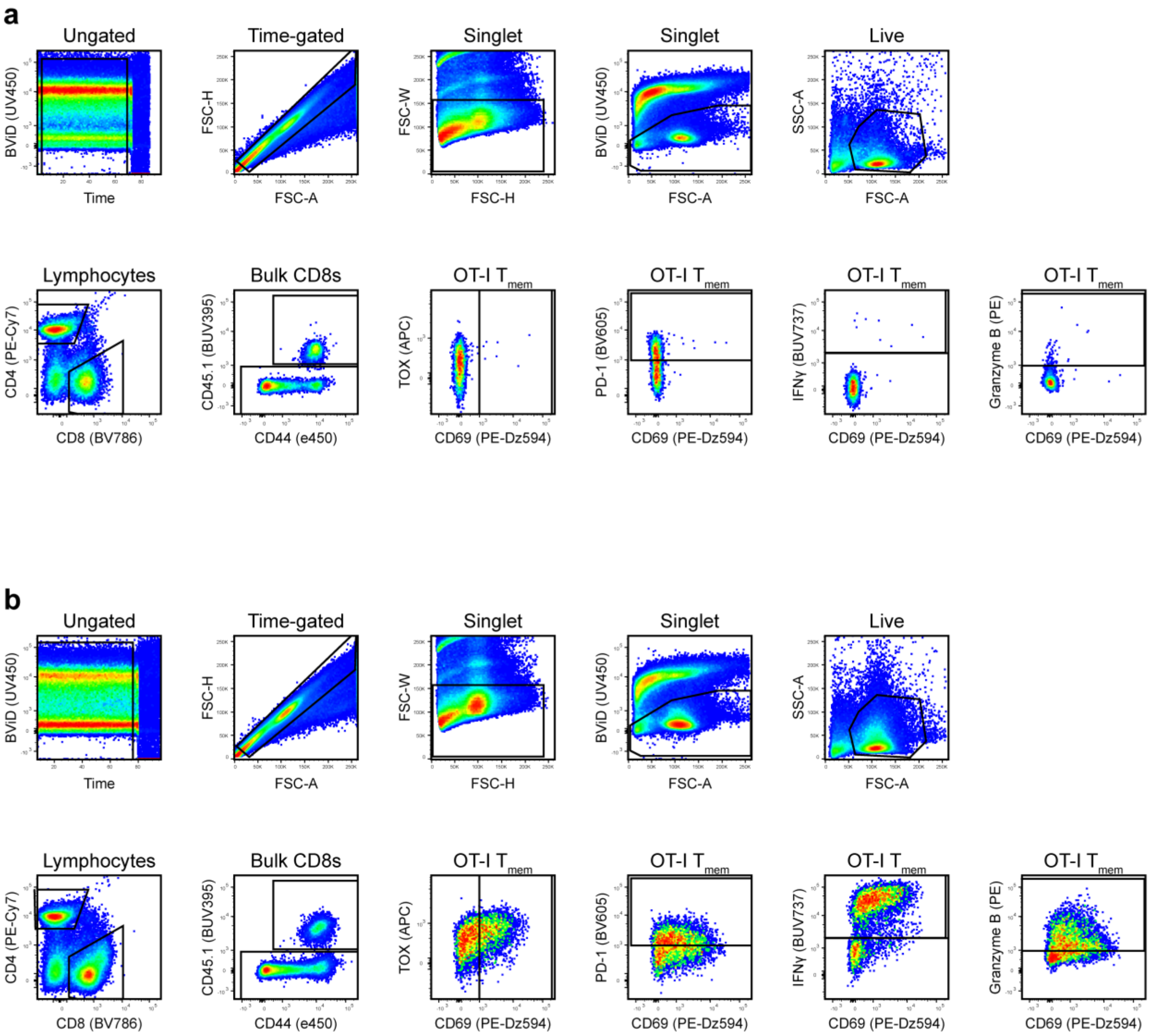

Supplemental Fig. 9. related to Fig. 2, Supplemental Fig. 2

a and **b** Representative flow gating for intracellular cytokine staining in parallel with TOX and PD-1 interrogation in mouse T cells (**a** mock-stimulated, 24h; **b** IL-12/15/18-stimulated, 24h).

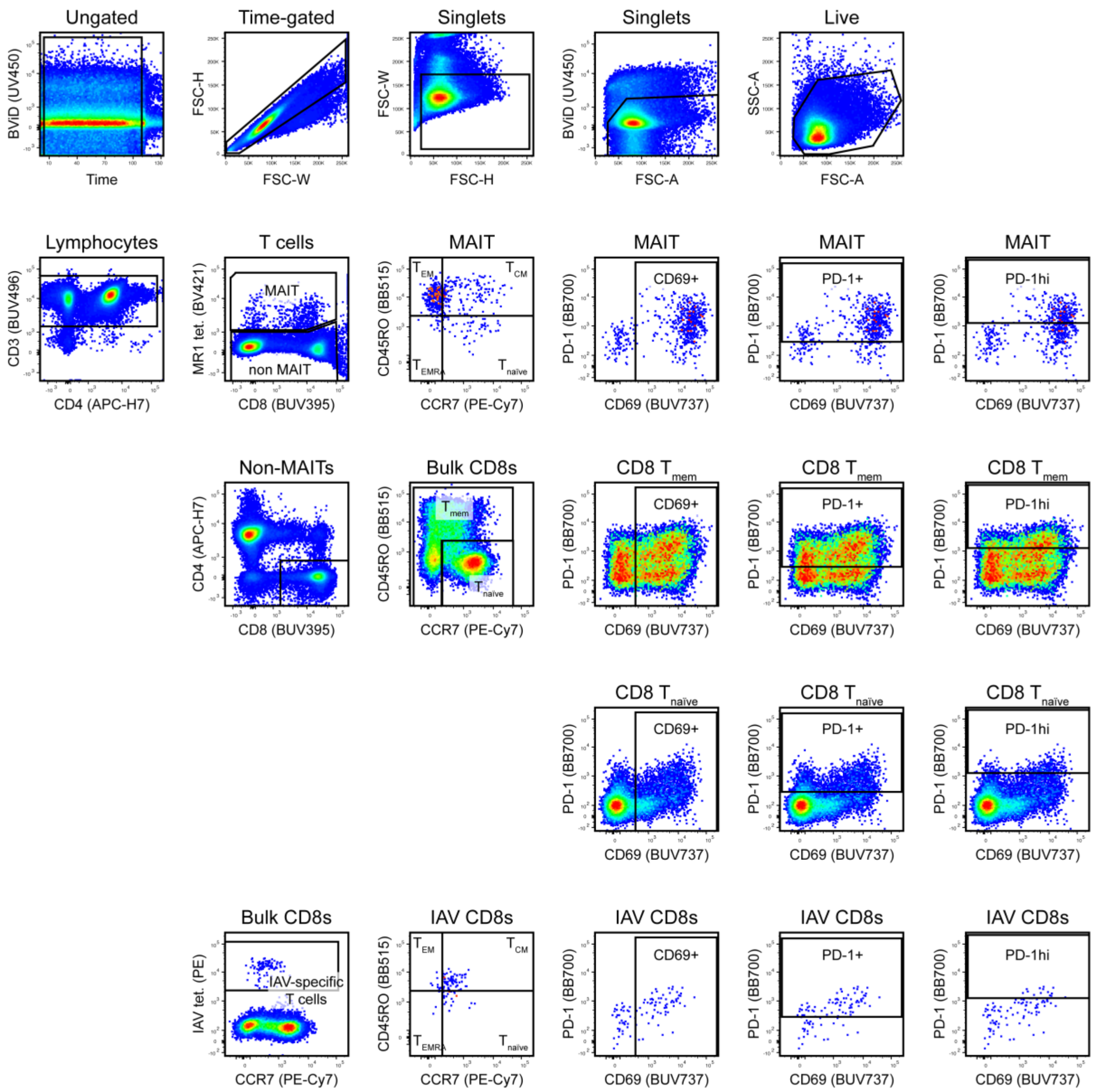

Supplemental Fig. 10. related to Fig. 4, 5, 6, Supplemental Fig. 4, 5, 6

Representative flow gating for TOX and PD-1 interrogation in human T cells with IAV and MR1 tetramers (IL-12/15/18-stimulated, 24h).

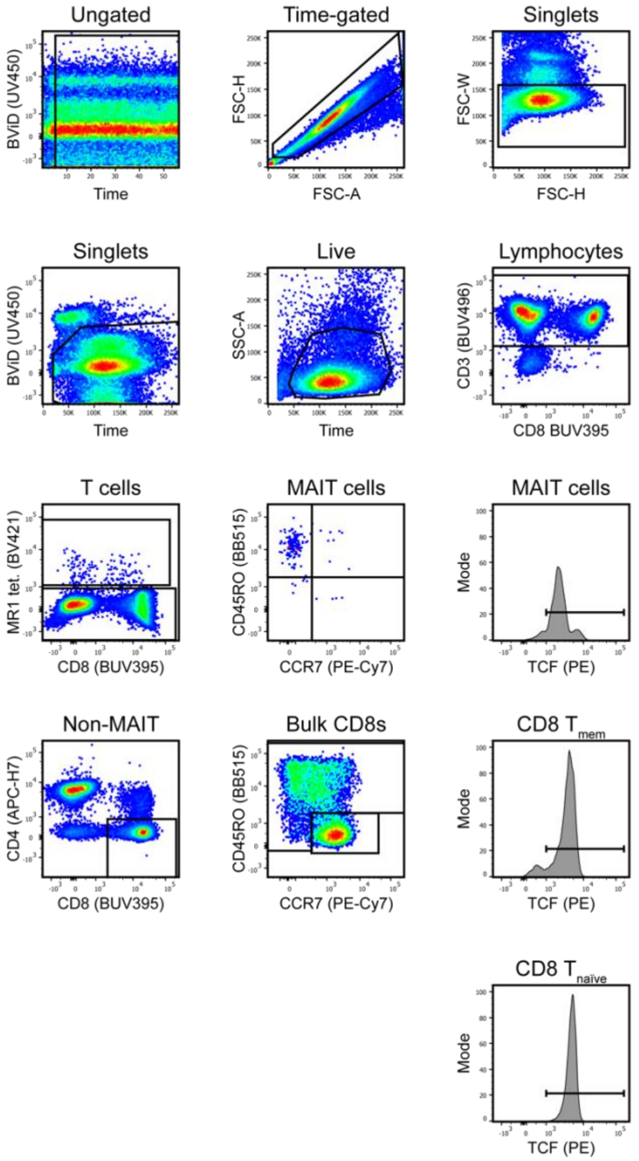

Supplemental Fig. 11. related to Fig. 5, Supplemental Fig. 4, 5

Representative flow gating for TOX, PD-1, and TCF1 interrogation in human T cells (unstimulated, 0h).

Reagent	Fluor	Clone	Vendor	Dilution
Viability stain (1x PBS diluent, 20 min, on ice)				
Zombie NIR fixable viability kit (NIRViD)	APC-Cy7	NA	BioLegend	1:500
Surface stain (FACSWash diluent, 30 min, on ice)				
Fc block (CD16/CD32)	Unconjugated	2.4G2	BD	1:200
CD4	PerCP-Cy5.5	RM4-4	BioLegend	1:200
CD8α	AF700	53-6.7	Thermo Fisher	1:200
CD45.1	PE-Cy7	A20	Thermo Fisher	1:200
CD44	BV421	IM7	BioLegend	1:200
PD-1	BV605	29F.1A12	BioLegend	1:100
CD62L	BV785	MEL-14	BioLegend	1:200
Fix (1x eBioscience FOXP3 fixation buffer, 20 min, on ice)				
Intracellular stain (1x eBioscience FOP3 permeabilizaiton buffer diluent, 30 min, on ice)				
Fc block (CD16/32)	Unconjugated	2.4G2	BD	1:200
TOX	e660	TXRX10	Thermo Fisher	1:200
TCF1/7	PE	S33966	BD	1:200

Supplemental Table 1. Mouse flow cytometry panel for SPF C57BL/6J, LCMV Armstrong P14 memory mice, and LCMV Docile P14 memory mice

Reagent	Fluor	Clone	Vendor	Dilution
Viability stain (1x PBS diluent, 20 min, on ice)				
LIVE/DEAD fixable blue viability dye (BViD)	UV450	NA	Thermo Fisher	1:500
Surface stain (FACSWash diluent, 30 min, on ice)				
Fc block (CD16/CD32)	Unconjugated	2.4G2	BD	1:200
CD4	BV786	GK1.5	BD	1:200
CD8α	PE-CF594	53-6.7	BD	1:200
CD45.1	BUV395	A20	BD	1:200
CD44	e450	IM7	Thermo Fisher	1:200
PD-1	BV605	29F.1A12	BioLegend	1:100
CD62L	FITC	MEL-14	Thermo Fisher	1:200
Fix (1x eBioscience FOXP3 fixation buffer, 20 min, on ice)				
Intracellular stain (1x eBioscience FOP3 permeabilizaiton buffer diluent, 30 min, on ice)				
Fc block (CD16/32)	Unconjugated	2.4G2	BD	1:200
TOX	APC	REA473	Miltenyi Biotec	1:100
TCF1/7	PE	C63D9	Cell Signaling	1:40

Supplemental table 2. Mouse flow cytometry panel for VSV-OVA OT-I memory mice, LM-OVA OT-I memory mice, and LM-gB gBT-I memory mice.

Reagent	Fluor	Clone	Vendor	Dilution
Viability stain (1x PBS diluent, 20 min, on ice)				
LIVE/DEAD fixable aqua viability dye (AViD)	V510	NA	Thermo Fisher	1:500
Surface stain (FACSWash diluent, 30 min, on ice)				
Fc block (CD16/CD32)	Unconjugated	2.4G2	BD	1:200
CD4	BV786	GK1.5	BD	1:200
CD8α	PE-CF594	53-6.7	BD	1:200
CD45.1	BUV395	A20	BD	1:200
CD45.2	FITC	104	Thermo Fisher	1:200
CD44	APC-e780	IM7	Thermo Fisher	1:200
PD-1	BV605	29F.1A12	BioLegend	1:100
Fix (1x eBioscience FOXP3 fixation buffer, 20 min, on ice)				
Intracellular stain (1x eBioscience FOP3 permeabilizaiton buffer diluent, 30 min, on ice)				
Fc block (CD16/32)	Unconjugated	2.4G2	BD	1:200
TOX	APC	REA473	Miltenyi Biotec	1:100

Supplemental table 3. Mouse flow cytometry panel for VSV-OVA OT-I memory mice.

Reagent	Fluor	Clone	Vendor	Dilution
Viability stain (1x PBS diluent, 20 min, on ice)				
LIVE/DEAD fixable blue viability dye (BViD)	UV450	NA	Thermo Fisher	1:500
Surface stain (FACSWash diluent, 30 min, on ice)				
Fc block (CD16/CD32)	Unconjugated	2.4G2	BD	1:200
CD4	PE-Cy7	RM4-5	BD	1:200
CD8α	BV786	53-6.7	BD	1:200
CD44	e450	IM7	Thermo Fisher	1:200
CD62L	FITC	MEL-14	Thermo Fisher	1:200
CD69	PE-Dazzle594	H1.2F3	BioLegend	1:100
NKG2D	BV711	CX5	BD	1:200
PD-1	BV605	29F.1A12	BioLegend	1:100
Fix (1x eBioscience FOXP3 fixation buffer, 20 min, on ice)				
Intracellular stain (1x eBioscience FOP3 permeabilizaiton buffer diluent, 30 min, on ice)				
Fc block (CD16/32)	Unconjugated	2.4G2	BD	1:200
IFNγ	BUV737	XMG1.2	BD	1:200
Granzyme B	PE	GB11	Thermo Fisher	1:100
TOX	APC	REA473	Miltenyi Biotec	1:100

Supplemental table 4. Mouse ICS flow cytometry panel for VSV-OVA OT-I memory mice.

Reagent	Fluor	Clone	Vendor	Dilution
Viability stain (1x PBS diluent, 20 min, room temperature)				
LIVE/DEAD fixable blue viability dye (BViD)	UV450	NA	Thermo Fisher	1:500
Tetramer stain (FACSWash diluent, 60 min, room temperature)				
MR1 OP-5-RU (MAIT cell) tetramer	BV421	NA	NIH Tetramer Core	1:500
HLA-A*02 influenza A virus (IAV) (GILGFVFTL) tetramer	PE	NA	Fred Hutch Immune Monitoring	1:150
TruStain FcX (Fc block)	NA	NA	BioLegend	1:20
Surface stain (FACSWash diluent, 20 min, room temperature)				
CD3	BUV496	UCHT1	BD	1:40
CD4	APC-H7	RPA-T4	BD	1:40
CD8	BUV395	RPA-T8	BD	1:80
CD45RO	BB515	UCHL1	BD	1:160
CD69	BUV737	FN50	BD	1:40
CCR7	PE-Cy7	3D12	BD	1:40
PD-1	BB700	EH12.1	BD	1:20
Fix (1x eBioscience FOXP3 fixation buffer, 20 min, room temperature)				
Intracellular stain (1x eBioscience FOP3 permeabilizaiton buffer diluent, 30 min, room temperature)				
TOX*	APC	REA473	Miltenyi Biotec	1:80
KLH-specific REA control antibody I*	APC	REA293	Miltenyi Biotec	1:80

Supplemental table 5. Flow cytometry panel for HLA-A*02 human PBMC samples.

*Reagents were not used in unison, but as stains in separate samples.

Reagent	Fluor	Clone	Vendor	Dilution
Viability stain (1x PBS diluent, 20 min, room temperature)				
LIVE/DEAD fixable blue viability dye (BViD)	UV450	NA	Thermo Fisher	1:500
Tetramer stain (FACSWash diluent, 60 min, room temperature)				
MR1 OP-5-RU (MAIT cell) tetramer	BV421	NA	NIH Tetramer Core	1:500
TruStain FcX (Fc block)	NA	NA	BioLegend	1:20
Surface stain (FACSWash diluent, 20 min, room temperature)				
CD3	BUV496	UCHT1	BD	1:40
CD4	APC-H7	RPA-T4	BD	1:40
CD8	BUV395	RPA-T8	BD	1:80
CD45RO	BB515	UCHL1	BD	1:160
CD69	BUV737	FN50	BD	1:40
CCR7	PE-Cy7	3D12	BD	1:40
PD-1	BB700	EH12.1	BD	1:20
Fix (1x eBioscience FOXP3 fixation buffer, 20 min, room temperature)				
Intracellular stain (1x eBioscience FOP3 permeabilizaiton buffer diluent, 30 min, room temperature)				
TOX	APC	REA473	Miltenyi Biotec	1:80
TCF1/7	PE	C63D9	Cell Signaling	1:40

Supplemental table 6. Flow cytometry panel for general human PBMC samples.
